# Integrated stem cell signature and cytomolecular risk determination in pediatric acute myeloid leukemia

**DOI:** 10.1038/s41467-022-33244-6

**Published:** 2022-09-19

**Authors:** Benjamin J. Huang, Jenny L. Smith, Jason E. Farrar, Yi-Cheng Wang, Masayuki Umeda, Rhonda E. Ries, Amanda R. Leonti, Erin Crowgey, Scott N. Furlan, Katherine Tarlock, Marcos Armendariz, Yanling Liu, Timothy I. Shaw, Lisa Wei, Robert B. Gerbing, Todd M. Cooper, Alan S. Gamis, Richard Aplenc, E. Anders Kolb, Jeffrey Rubnitz, Jing Ma, Jeffery M. Klco, Xiaotu Ma, Todd A. Alonzo, Timothy Triche, Soheil Meshinchi

**Affiliations:** 1 Department of Pediatrics, University of California San Francisco, San Francisco, CA USA; 2 Helen Diller Family Comprehensive Cancer Center, University of California San Francisco, San Francisco, CA USA; 3 Fred Hutchinson Cancer Research Center, Seattle, WA USA; 4 University of Arkansas for Medical Sciences & Arkansas Children’s Research Institute, Little Rock, AR USA; 5 Children’s Oncology Group, Monrovia, CA USA; 6 Department of Pathology, St. Jude Children’s Research Hospital, Memphis, TN USA; 7 Nemours Center for Cancer and Blood Disorders and Alfred I. DuPont Hospital for Children, Wilmington, DE USA; 8 Division of Hematology/Oncology, Seattle Children’s Hospital, University of Washington, Seattle, WA USA; 9 School of Medicine, University of California, San Francisco, San Francisco, CA USA; 10 Department of Computational Biology, St. Jude Children’s Research Hospital, Memphis, TN USA; 11 Michael Smith Genome Sciences Centre, Vancouver, BC Canada; 12 Children’s Mercy Hospitals and Clinics, Kansas City, MO USA; 13 Children’s Hospital of Philadelphia, Philadelphia, PA USA; 14 Department of Oncology, St. Jude Children’s Research Hospital, Memphis, TN USA; 15 Department of Preventive Medicine, University of Southern California, Los Angeles, CA USA; 16 Van Andel Research Institute, Grand Rapids, MI USA

**Keywords:** Acute myeloid leukaemia, Prognostic markers, Gene expression, Acute myeloid leukaemia

## Abstract

Relapsed or refractory pediatric acute myeloid leukemia (AML) is associated with poor outcomes and relapse risk prediction approaches have not changed significantly in decades. To build a robust transcriptional risk prediction model for pediatric AML, we perform RNA-sequencing on 1503 primary diagnostic samples. While a 17 gene leukemia stem cell signature (LSC17) is predictive in our aggregated pediatric study population, LSC17 is no longer predictive within established cytogenetic and molecular (cytomolecular) risk groups. Therefore, we identify distinct LSC signatures on the basis of AML cytomolecular subtypes (LSC47) that were more predictive than LSC17. Based on these findings, we build a robust relapse prediction model within a training cohort and then validate it within independent cohorts. Here, we show that LSC47 increases the predictive power of conventional risk stratification and that applying biomarkers in a manner that is informed by cytomolecular profiling outperforms a uniform biomarker approach.

## Abstract (web summary)

Relapsed pediatric acute myeloid leukemia is associated with poor prognosis. Here, the authors use RNA-seq data from 1503 primary samples to create a combined transcriptional and cytomolecular signature to improve relapse risk prediction.

## Introduction

Acute myeloid leukemia (AML) remains a therapeutic challenge with high mortality rates despite intensive and myeloablative therapies^1,2^. Structural and sequence alterations have been linked to outcomes in AML and have been used for risk-based therapy allocation with modest success^3–10^. However, given the vast heterogeneity of AML, conventional cytogenetic and molecular (cytomolecular) biomarkers have not yielded a robust prognostic model to date. Specifically, nearly one-third of pediatric patients classified as “low risk” ultimately relapse. Conversely, approximately one-third of those in “high risk” categories have favorable outcomes. An AML study in adults previously identified 47 unique genes enriched in leukemia stem cell populations (LSC47) and extracted an optimal 17 gene signature (LSC17) that was highly prognostic across five independent cohorts comprised of adult patients with diverse AML subtypes (*n* = 908)^11^. We hypothesized that incorporating a similar scoring system in pediatric AML would lead to improved prognostic risk models.

Here, we describe how LSC17 and related LSC signatures impact risk prediction for de novo AML diagnosed in children, adolescents, and young adults (*n* = 1503). While LSC17 has been previously studied in pediatric cohorts^12,13^, these analyses were limited by relatively small samples sizes (*n* = 371 and *n* = 368, respectively) that precluded robust comparisons with established cytomolecular risk models and other biomarkers. Our data supports a significant association between LSC17 and cytomolecular risk stratification and underlying gene fusions. Additionally, we demonstrate that a “one size fits all” approach does not capture the heterogeneity across a large cohort of pediatric AMLs and fails to leverage the similarities within cytomolecular subgroups that drive leukemia biology, biomarker statistical significance, and ultimately survival. Additionally, we propose a robust risk prediction model that applies a pediatric LSC signature (LSC47) based on the 47 upregulated LSC gene set and informed by underlying cytomolecular alteration, and we demonstrate that this integrated approach is superior to either LSC17 or cytomolecular risk stratification alone.

## Results

### LSC17 in pediatric AML

**Fig. 1:**
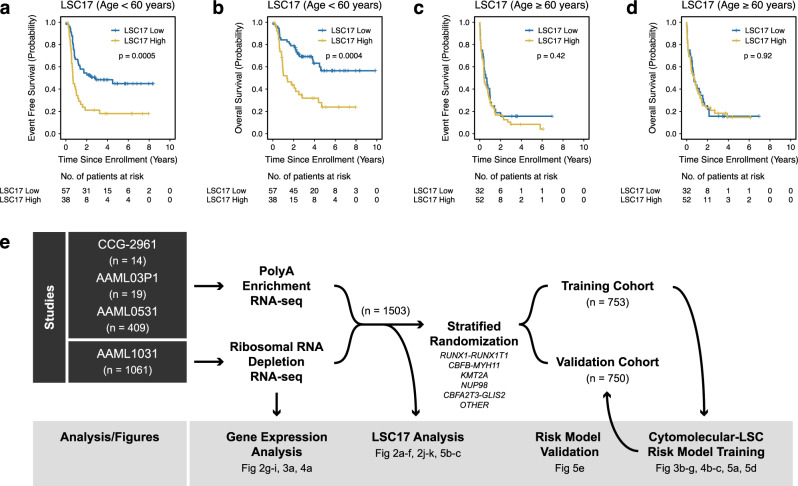
LSC17 in TCGA AML based on age. **a**–**d** Kaplan–Meier estimates for the probability of event free survival (EFS) and overall survival (OS) in patients from The Cancer Genome Atlas (TCGA) AML cohort segregated based on age. **a**, **b** LSC17 scores predict survival in younger adults (<60 years of age). **c**, **d** Conversely, LSC17 scores do not discriminate between favorable and unfavorable outcomes in older adults (≥60 years of age). Survival differences were determined using the log-rank test (two-sided and without multiple-testing adjustments). **e** Schematic diagram for our experimental design. Our data set consists of primary samples that were obtained at the time of diagnosis after enrollment in one of four clinical trials listed in the left panel (black). Specific data analyses and associated figures are noted in the bottom panel (gray). Samples underwent either polyadenylation enrichment or ribosomal RNA depletion. Stratified randomization was performed based on fusion category to generate two cohorts for risk model training and validation.

**Fig. 2:**
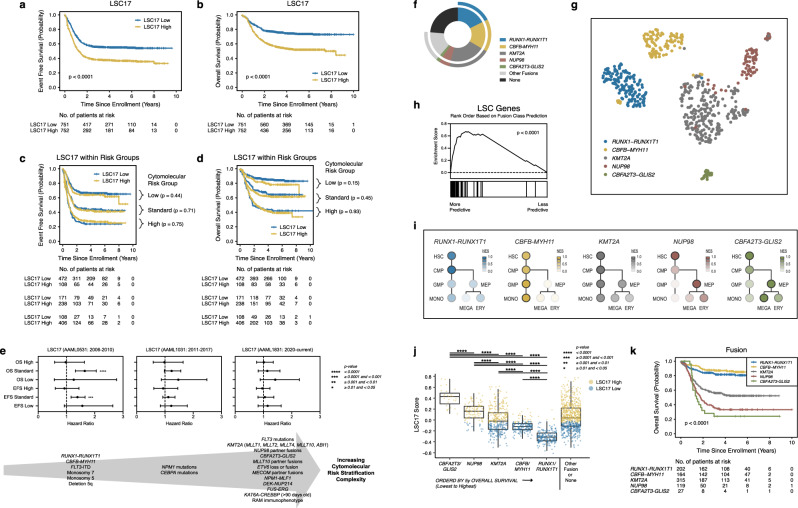
LSC17 in pediatric AML. Kaplan–Meier estimates for the probability of **a** EFS and **b** OS in patients within our entire cohort (*n* = 1503) stratified based on low versus high LSC17 scores. LSC17 scores significantly predict survival for the entire non-stratified cohort. Conversely, LSC17 scores do not improve upon previously established risk stratification models based on cytogenetic and molecular alterations in regards to either **c** EFS or **d** OS. **e** Hazard ratios with 95% confidence intervals for EFS and OS as a function of LSC17 risk group (high versus low) across historical clinical trial cytomolecular risk stratification schema (*n* = 1503 patients). **f** Driver gene fusion frequencies within our entire study cohort (*n* = 1503). **g** Uniform manifold approximation and projection (UMAP) performed on selected genes based on the nearest shrunken centroids approach clearly discriminates fusion classes. **h** Gene set enrichment analysis on a 47 LSC gene signature reveals that LSC genes are significantly enriched among fusion-predictive genes. GSEA *p*-values are calculated by permutation (*n* = 1000) across the gene set of interest combined with every gene set within the Broad Institute Molecular Signature Database v6.2. **i** Normalized enrichment scores based on hematopoietic hierarchical cell populations reveal that gene fusion transcriptional signatures align with distinct hematopoietic stem cell and myeloid progenitor cell population states. NES normalized enrichment score. **j** Box plot of LSC17 scores categorized based on cytogenetic or fusion status reveal that LSC17 scores significantly correlate with underlying alteration (*n* = 1503 patients). Box plot data are presented as median values with hinges corresponding to the 25th or 75th percentiles and whiskers corresponding to 1.5 times the inter-quartile range. *P*-values were calculated based on two-sided t-tests. Source data are provided as a Source Data file. **k** Survival outcomes stratified based on fusion status. Survival differences were determined using the log-rank test (two-sided and without multiple-testing adjustments).

To assess the impact of age on LSC17 score prediction, we analyzed the TCGA AML patient cohort. While LSC17 scores were predictive of survival for patients diagnosed at less than 60 years of age, they were not predictive for those diagnosed at greater than or equal to 60 years of age (Fig. 1a–d). While consistent with previous observations that both age and LSC17 scores retained significant prognostic value in multivariable survival analysis of adult AML^11^, these data also suggested that it would be valuable to systematically investigate LSC17 in younger patients (diagnosed at less than 30 years of age). Therefore, we harnessed RNA-sequencing performed in diagnostic AML samples from 1,503 patients enrolled in one of four clinical trials (Fig. 1e) to perform gene expression and LSC17 score analyses. We also divided our study population into training (*n* = 753) and validation (*n* = 750) cohorts (Table 1) stratified based on fusion status to generate a risk predication model and then subsequently validate the model, respectively. In aggregate, patients with a high LSC17 score had an event free survival (EFS) of 36.7 ± 3.6% at 5 years from diagnosis compared to 55.1 ± 3.7% for those with low LSC17 scores (*p* < 0.0001) (Fig. 2a). LSC17 scores were also associated with adverse overall survival (OS): 51.9 ± 3.9% versus 73.7 ± 3.4% (*p* < 0.0001) (Fig. 2b). We then evaluated LSC17 scores in the context of established cytomolecular risk stratification and found that LSC17 scores were no longer predictive of survival within low, standard, or high-risk groups (Fig. 2c, d). This observation is also true if we reassigned LSC17 category (low versus high) based on the median score within a given cytomolecular risk group (Supplementary Fig. 1). Additionally, the ability for LSC17 to predict outcome decreases with increasing cytomolecular risk stratification complexity that occurred over a series of clinical trials conducted over the past 15 years (Fig. 2e). Since age of diagnosis plays an important role for LSC17 within the TCGA AML patient cohort (Fig. 1a), we asked whether age-based differences play a role within our pediatric cohort. Categorizing patients based on age (children, ages 0–10 years; adolescents, ages 10–18 years; and young adults, ages 18–30 years) resulted in similar findings as Fig. 2a–d. Specifically, while LSC17 scores were prognostic for EFS and OS in every age category, they were no longer predictive of survival within established cytomolecular risk groups (Supplementary Figs. 2–4).

**Table 1:** Patient characteristics

Characteristic	Training cohort (*n* = 753)	Validation cohort (*n* = 750)	*P*-value*
Sex			
Female	359 (47.7)	370 (49.3)	0.520
Male	394 (52.3)	380 (50.7)	
Age			
<3 years	175 (23.2)	174 (23.2)	0.985
3–5 years	60 (8.0)	63 (8.4)	0.760
5–10 years	134 (17.8)	145 (19.3)	0.443
10–18 years	323 (42.9)	318 (42.4)	0.846
>18 years	61 (8.1)	50 (6.7)	0.288
WBC Count			
<100,000/µL	594 (79.0)	586 (78.1)	0.686
≥100,000/µL	158 (21.0)	164 (21.9)	
Unknown	1	0	
Cytomolecular risk group			
Low	291 (38.6)	289 (38.5)	0.964
Standard	212 (28.2)	197 (26.3)	0.411
High	250 (33.2)	264 (35.2)	0.414
MRD at end of induction I			
No	462 (68.8)	468 (69.0)	0.796
Yes	210 (31.3)	210 (31.0)	
Unknown	81	72	
SCT in CR1			
No	650 (86.3)	659 (87.9)	0.372
Yes	103 (13.7)	91 (12.1)	
*CEBPA* mutation			
No	713 (94.7)	707 (94.3)	0.721
Yes	40 (5.3)	43 (5.7)	
*FLT3*-ITD mutation			
No	609 (80.9)	602 (80.3)	0.765
<0.1	26 (3.5)	28 (3.7)	0.770
≥0.1	118 (15.7)	120 (16.0)	0.861
Fusion category			
* RUNX1-RUNX1T1*	101 (13.4)	101 (13.5)	0.976
* CBFB-MYH11*	82 (10.9)	82 (10.9)	0.978
* KMT2A*	158 (21.0)	157 (20.9)	0.981
* NUP98*	60 (8.0)	59 (7.9)	0.942
* CBFA2T3-GLIS2*	14 (1.9)	13 (1.7)	0.854
Other or no fusion	338 (44.9)	338 (45.1)	0.944

Demographic and molecular characteristics of our study cohort. Abbreviations include *WBC* white blood cells, *CNS* central nervous system, *MRD* minimal residual disease, *SCT* stem cell transplant, *CR1* first complete remission, *ITD* internal tandem duplication, *KD* kinase domain. *P*-values were based on the chi-squared test.

### Gene fusions are linked to distinct transcriptional signatures

Since cytomolecular risk stratification in pediatric AML is driven, in large part, by recurring gene fusions, we then analyzed the impact of fusions on leukemia stem cell signatures. We grouped patients based on the presence of one of five gene fusion classes, which represent 55.0% of the AMLs within the overall cohort (*RUNX1-RUNX1T1*, *CBFB-MYH11*, *KMT2A* partner fusions, *NUP98* partner fusions, and *CBFA2T3-GLIS2*) (Fig. 2f). In this analysis, LSC17 scores were only predictive of survival in the *KMT2A* and “Other or No Fusion” (defined as not containing one of the five gene fusion classes) AML subgroups, but not predictive of survival in the *RUNX1-RUNX1T1*, *CBFB-MYH11*, *NUP98*, and *CBFA2T3-GLIS2* subgroups (Supplementary Fig. 5). A core subset of genes identified using the nearest shrunken centroids approach^14^ clearly discriminates gene fusion positive AMLs from one another (Fig. 2g and Supplementary Fig. 6) and 47 LSC upregulated genes identified by Ng, et al.^11^ (LSC47) are enriched among more predictive fusion class genes (Fig. 2h). Additionally, gene fusion positive AMLs are enriched in transcriptional signatures that mirror distinct hematopoietic stem cell and myeloid progenitor cell population states (Fig. 2i and Supplementary Fig. 7), which is consistent with previous findings^15–24^. Specifically, *CBFA2T3-GLIS2* AMLs expressed megakaryocytic transcriptional signatures^15–17^; *NUP98* partner fusion AMLs expressed erythroid transcriptional signatures^18,19^, *CBFB-MYH11* and *KMT2A* partner fusion AMLs expressed myelomonocytic transcriptional signatures^20–22^, and *RUNX1-RUNX1T1* AMLs expressed myeloblastic transcriptional signatures^23,24^.

### LSC17 Scores Cluster Based on Fusion

Since gene fusions are linked to distinct transcriptional signatures, we next determined the relationship between LSC17 scores and AML gene fusions. Intriguingly, we found that LSC17 scores significantly cluster within fusion classes (Fig. 2j) and that median LSC17 scores for a given fusion class closely correlates with survival based on fusion status (Fig. 2k). Additionally, while *KMT2A* fusion positive AMLs are associated with a large variance, LSC17 scores also significantly cluster based on the associated *KMT2A* gene partners, *FLT3* internal tandem duplication (ITD) status, and cytomolecular risk group (Supplementary Fig. 8).

### Cytomolecular-specific LSC signatures

**Fig. 3:**
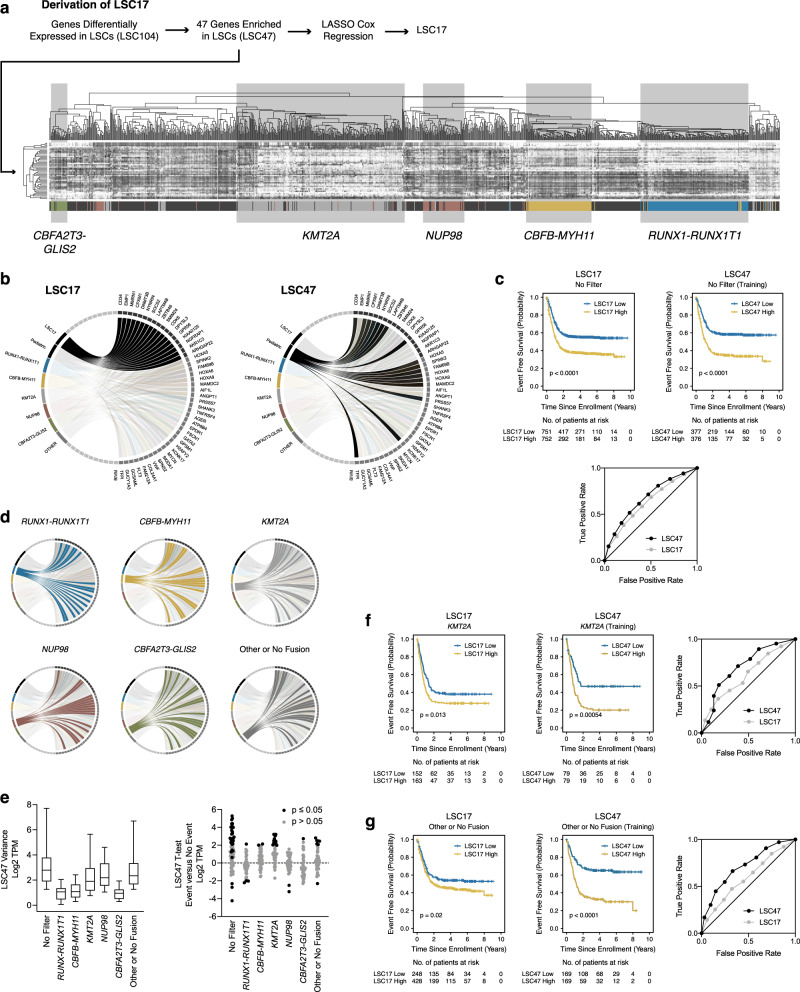
Leukemia stem cell transcriptional signature for pediatric AML. **a** The LSC17 gene signature was previously generated based on LASSO Cox regression on 47 genes enriched in LSC AML cell populations (LSC47). Analyzing LSC47 gene expression data within our cohort, AMLs cluster based on underlying fusion category. **b** The circos plot on the left indicates the previously described LSC17 gene set. Conversely, the circos plot on the right indicates the 17 most predictive genes within our training cohort using the same LASSO based Cox regression analysis. Subsequent risk stratification model building considers all 47 upregulated LSC genes (LSC47). **c** Kaplan–Meier estimates for the probability of EFS based on LSC17 versus LSC47 gene signatures and associated area under the curve receiver operating characteristic (AUC ROC) curve plotting true positive rates versus false positive rates as a function of LSC17 and LSC47 score thresholds. **d** Additionally, when AMLs are grouped based on underlying fusion, each class is associated with a distinct LSC gene set. **e** LSC47 variance and t-tests based on fusion category (*n* = 753 patients from the training cohort). Box plot data are presented as median values with hinges corresponding to the 25th or 75th percentiles and whiskers corresponding to the 10th or 90th percentiles (left panel). *P*-values were calculated based on two-sided t-tests (right panel). Source data are provided as a Source Data file. Kaplan–Meier estimates for the probability of EFS and AUC ROC curves among **f** *KMT2A* and **g** Other or No Fusion AML cohorts based on LSC17 versus LSC47. Survival differences were determined using the log-rank test (two-sided and without multiple-testing adjustments).

Hierarchical clustering on LSC47 within our cohort differentiated the five core fusion classes (Fig. 3a). The same study^11^ that experimentally identified LSC47, which represents 47 genes differentially upregulated in LSC+ populations, also identified a broader gene set (LSC104) that also includes genes that are differentially downregulated in LSC+ populations. Repeating hierarchical clustering on LSC104 within our cohort is neither more sensitive nor specific in differentiating fusion classes (Supplementary Fig. 9). Based on our LSC47 findings, we asked whether LSC gene expression data could be utilized to generate a more robust risk classification schema in the context of specific structural variants. The Children’s Oncology Group (COG) study population was divided into training (*n* = 753) and validation (*n* = 750) cohorts stratified based on fusion status (*RUNX1-RUNX1T1*, *CBFB-MYH11*, *KMT2A*, *NUP98*, *CBFA2T3-GLIS2*, and Other or No Fusion) (Fig. 1e and Table 1). To develop more predictive biomarkers related to stemness, we used LSC47 to perform the same analysis that generated the original LSC17 signature. Specifically, we performed linear regression based on the LASSO algorithm to fit a Cox regression model using LSC47 within our training cohort. This analysis revealed a distinct and more predictive gene signature for our training cohort, which we designate LSC47 (Fig. 3b, c and Supplementary Table 1). Performing the same analysis within each fusion class, we again identified distinct LSC gene signatures for each class (Fig. 3d). Since gene expression variances and t-tests differed based on fusion class (Fig. 3e), we performed internal cross validation analysis within the training cohort fusion subgroups by iteratively and randomly dividing subgroups in half and repeating the LASSO Cox regression modeling analysis to determine whether iterative gene signatures remain predictive in the non-modeled half (Supplementary Fig. 10). LSC gene signatures specifically remained predictive within for *KMT2A* and Other or No Fusion AML subgroups (Fig. 3f, g) and the associated coefficients are included in Supplementary Table 1.

**Fig. 4:**
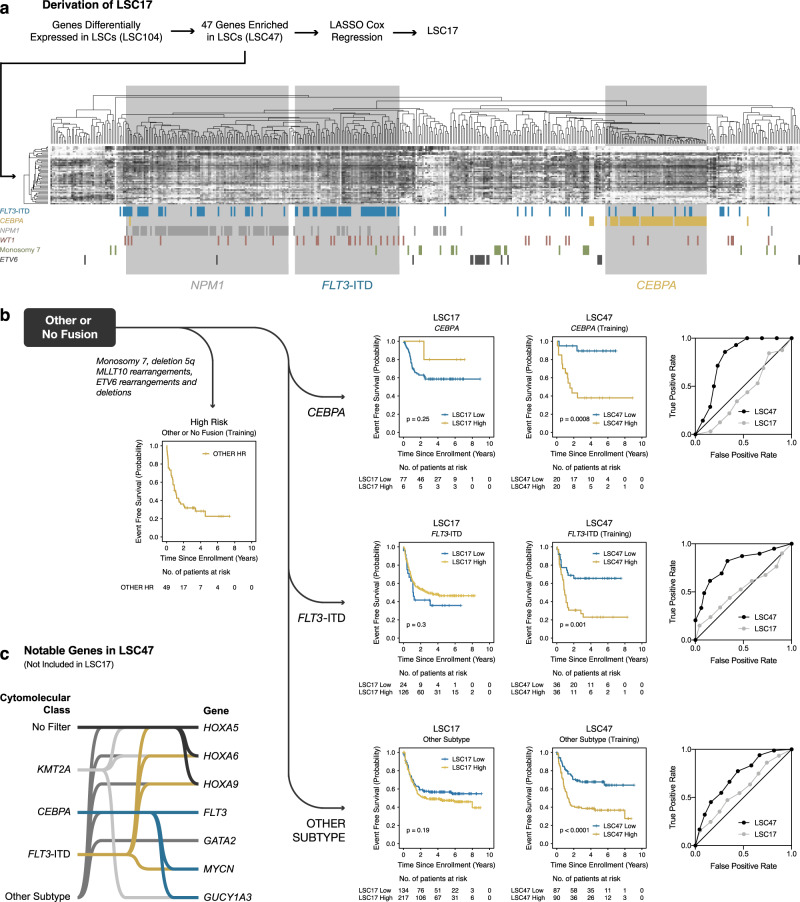
Additional transcriptional biomarkers for pediatric AML. **a** Isolating AMLs that did not have one of the five core fusion alterations, additional cytomolecular subtypes clustered with one another based on LSC47: *NPM1*, *CEBPA*, and *FLT3* internal tandem duplication (ITD) mutation. **b** Kaplan–Meier estimates for the probability of EFS within the training cohort for *CEBPA*, *FLT3*-ITD, and Other Subtype AMLs. Survival differences were determined using the log-rank test (two-sided and without multiple-testing adjustments). **c** Notable genes included in LSC47 but not LSC17. Genes are connected to the cytomolecular classes based on whether they contribute to the associated LSC signature and score.

Hierarchical clustering on LSC47 within AMLs within the Other or No Fusion AML subgroup resulted in additional cytomolecular subtype clustering in AMLs with *NPM1*, *CEBPA*, or *FLT3*-ITD mutations (Fig. 4a). Again, performing LASSO Cox regression using the LSC47 and internal cross validation analysis within these subsets yielded predictive signatures for *CEBPA* mutated, *FLT3*-ITD, and Other Subtype (defined as not containing a core gene fusion, *CEBPA* mutation, or *FLT3*-ITD) subgroups (Fig. 4b). Based on previous leukemia biology studies, notable genes included in these gene signatures that are not included in LSC17 are *HOXA5*^25–28^, *HOXA6*^25,29^, *HOXA9*^25,28–31^, *FLT3*^32–35^, *GATA2*^36,37^, *MYCN*^38,39^, and *GUCY1A3*^40,41^ (Fig. 4c).

To differentiate favorable versus less favorable risk core-binding factor (CBF) AMLs (i.e., *RUNX1-RUNX1T1* and *CBFB-MYH11*), we implemented previously published biomarkers. For *RUNX1-RUNX1T1* AMLs, a previously described *RUNX1* transcriptional signature^42^ was predictive within our training cohort (Supplementary Fig. 11). For *CBFB-MYH11* AMLs, fusion breakpoint location^21^ nearly reached significance in prediction of EFS within our training cohort (Supplementary Fig. 11) and reached significance within our previously published results when the *CBFB-MYH11* AML cohort was analyzed in aggregate^43^.

### Integrated stem cell signature and cytomolecular risk determination model

**Fig. 5:**
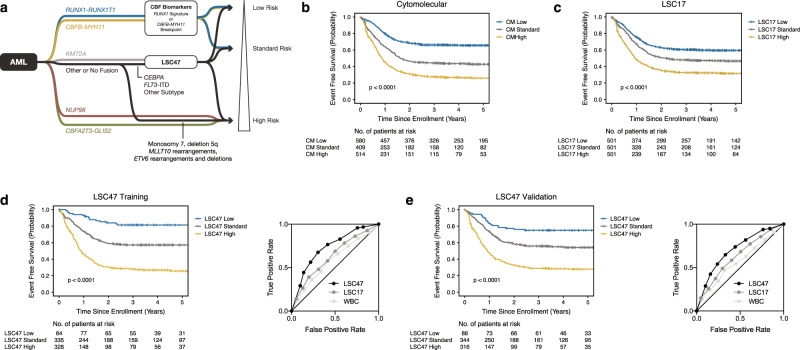
LSC47 risk stratification model. **a** To build a robust risk prediction model for pediatric AML, we aggregated LSC47 based signatures with other validated biomarkers (e.g., *RUNX1-RUNX1T1* transcriptional signature and *CBFB-MYH11* fusion breakpoint location) within our training cohort. *NUP98* partner fusion and *CBFA2T3-GLIS2* AMLs are associated with 5-year EFS of < 20% and were therefore assigned to the high-risk stratum without further stratification. Kaplan–Meier estimates for the probability of EFS based on **b** cytomolecular (CM) risk factors, **c** LSC17, and **d**, **e** combined LSC47 model in training and validation cohorts. Survival differences were determined using the log-rank test (two-sided and without multiple-testing adjustments).

To build a robust risk prediction model for pediatric AML, we aggregated LSC47 signatures with the “best in class” biomarkers within each cytomolecular subtype (Fig. 5a). Since five-year EFS probabilities for *NUP98* and *CBFA2T3-GLIS2* AMLs are less than 20% in our cohort and previous studies^15,17,44^, no further risk stratification was attempted. Additional cytomolecular subclasses within Other or No Fusion AMLs class were assigned to high-risk with no further risk stratification based on previous studies: AMLs with monosomy 7 or deletion 5q^45–47^, *MLLT10* partner fusions^48^, or *ETV6* partner fusions and deletions^49^. Finally, LSC47 was applied to *KMT2A* fusion positive, *CEBPA* mutated, *FLT3*-ITD and Other Subtype AMLs as summarized in Figs. 3 and 4. These cytomolecular based biomarkers were then combined to build a robust risk determination model (Fig. 5d and Supplementary Fig. 12) that was then subsequently validated in our independent validation cohort (Fig. 5e and Supplementary Fig. 13).

We then compared our final LSC47 model with previous risk stratification models within our pediatric AML cohort. Utilizing contemporary cytomolecular risk stratification to classify our entire cohort into low, standard, and high-risk cytomolecular groups revealed EFS proportions of 65.8 ± 4.0, 42.4 ± 5.0, and 26.2 ± 4.0%, respectively (Fig. 5b). Segregating the same cohort into terciles based on LSC17 scores results in low, standard, and high-risk LSC groups with associated EFS of 59.5 ± 4.6, 46.7 ± 4.7, and 31.6 ± 4.3%, respectively (Fig. 5c). Finally, our combined LSC47 model identified low, standard, and high-risk LSC47 groups with associated EFS of 81.4 ± 8.7, 57.2 ± 5.6, and 25.6 ± 5.0% for the training cohort (Fig. 5d). An independent analysis of the validation cohort confirmed these findings with EFS of 75.0 ± 9.2, 54.7 ± 5.7, and 28.0 ± 5.2% for low, standard, and high-risk patients, respectively (Fig. 5e).

We then performed univariable survival analysis within our validation cohort based on the following covariates: age; white blood cell count; presence of *FLT3*-ITD, *NPM1* mutation, or *CEBPA* mutation; LSC17 score; LSC47 score; and fusion gene partner (Supplementary Table 2). Covariates that were significant (*p*-value ≤ 0.05) were then combined to perform multivariable survival analysis (Supplementary Table 3). Whereas LSC17 was no longer significant in multivariable analysis, LSC47 and other additional cytomolecular-specific biomarkers either retained significance or were superior to LSC17.

We then tested our LSC47 model on an independent validation cohort. We harnessed RNA-sequencing performed in diagnostic AML samples from 212 patients enrolled on St. Jude’s AML08 clinical trial^50^. We performed gene expression, LSC17 score, and LSC47 model analyses. Similar to our previous results, LSC17 scores cluster within fusion classes and is not prognostic in the context of established cytomolecular risk factors (Supplementary Fig. 14). Conversely, LSC47 significantly improves upon LSC17 stratification and remains predictive of EFS within the *KMT2A* partner fusion, *CEBPA* mutated, and *FLT3*-ITD subgroups (Supplementary Fig. 14).

## Discussion

This study demonstrates that a 47 gene LSC signature (Supplementary Table 2) enhances risk prediction in the context of conventional cytomolecular risk stratification and retains predictive power in multivariable analysis. We found that LSC17 remains an important transcriptional signature that tracks closely with pediatric AMLs based on underlying fusion status, but that distinct LSC signatures informed by cytomolecular status better predict survival among patients with AMLs characterized by *KMT2A* fusions, *CEBPA* mutations, *FLT3*-ITDs, and Other Subtype AMLs (with other or no identifiable cytomolecular alteration).

Efforts to advance our understanding of molecular alterations in pediatric AML^3,4,15,17,22,44,51^ have revealed profound heterogeneity and improved understanding for how mutations and structural alterations impact treatment outcome. In the current study, we revealed that these molecular alterations share a close relationship with LSC gene expression, wherein LSC genes can accurately discriminate between AML molecular subtypes on the basis of unsupervised clustering alone. While intriguing, however, this same finding best explains why LSC17 does not augment traditional cytomolecular risk stratification in pediatric AML. Nevertheless, by harnessing the original LSC gene set reported by Ng et al.^11^, we found that LSC signatures based on underlying molecular alterations are more impactful than a “one size fits all” biomarker approach and remain prognostic in the setting of even complex cytomolecular risk stratification (Fig. 5). *CBFB-MYH11* fusion breakpoint location is another prime example that emphasizes the importance of identifying and applying biomarkers on the basis of underlying molecular alteration.

LSC17 has been previously studied within pediatric AML cohorts^12,13^ and a six gene subset signature has previously been proposed (LSC6)^13^. While LSC6 represents a promising transcriptional biomarker, we performed parallel analysis on LSC6 within our cohort, as was shown for LSC17 (Fig. 2a–d) and our results suggest that LSC6 shares similar drawbacks as LSC17. Specifically, while high LSC6 scores were associated with adverse EFS and OS within our entire cohort, LSC6 scores were no longer predictive of survival when evaluated within the context of established cytomolecular risk stratification—with EFS in standard-risk patients being a notable exception (Supplementary Fig. 15). Furthermore, LSC6 scores also cluster within fusion classes (Supplementary Fig. 15) similar to LSC17 scores (Fig. 2j). These findings overall suggest that all-encompassing transcriptional signatures that are agnostic to pre-existing cytomolecular stratification are at risk of encoding overlapping prognostic information with traditional biomarkers (e.g., mutations, copy number alterations, etc.), particularly those that influence transcriptional states (e.g., fusions).

The prognostic implication of *KMT2A* fusion partners has been well described by our group and others^22,52,53^. Notably, the LSC47 gene signature performs well in multivariable survival analysis when compared with *KMT2A* fusion partner risk (validation cohort analysis included in Supplementary Tables 2 and 3). Additionally, LSC47 and *KMT2A* fusion partner risk both remain significant when we consider our entire cohort, suggesting that integrating the LSC47 gene signature and *KMT2A* fusion partner risk groups further augments the prognostic modeling for patients with *KMT2A* fusion positive AML and additional studies are underway.

As the cost of sequencing continues to improve rapidly, it has become feasible to consider and evaluate larger (rather than smaller), more powerful biomarker gene sets for cancer prognostication. While LSC17 is a powerful approach to capture outcome measures across diverse cohorts of patients diagnosed with AML, molecularly informed biomarkers such as LSC47 could advance the overall goal of using personalized medicine to better inform treatment decisions.

## Methods

### Patient samples and RNA-sequencing

Pediatric AML biological samples were collected with informed consent from patients diagnosed with de novo AML and enrolled on Children’s Oncology Group (COG) trials CCG-2961 (NCT00002798)^2^, AAML03P1 (NCT00070174)^54^, AAML0531 (NCT00372593)^1^, or AAML1031 (NCT01371981)^55^. Each protocol was approved by the National Cancer Institute’s central institutional review board (IRB) and the local IRB at each participating institution. Patients and/or families provided informed consent or assent as appropriate. For CCG-2961, patients (0–21 years of age) were enrolled from 1996 to 2003 and were randomized to one of two chemotherapy regimens and then proceeded with hematopoietic cell transplantation (HCT) if a donor was available. For AAML03P1, patients (0–21 years of age) were enrolled from 2003 to 2005 and non-randomly assigned gemtuzumab ozogamicin in combination with conventional chemotherapy and proceeded with HSCT if a donor was available. For AAML0531, patients (0–29 years of age) were enrolled from 2006 to 2010 and then randomized to receive gemtuzumab ozogamicin in combination with conventional chemotherapy and proceeded with HCT in the setting of high-risk disease with an available donor. For AAML1031, patients (0–29 years of age) were enrolled from 2011 to 2017 and then randomized to receive bortezomib in combination with conventional chemotherapy, non-randomly assigned to receive sorafenib in the setting of *FLT3*-ITD AML, and proceeded with HCT in the setting of high-risk disease with an available donor. Total RNA derived from peripheral blood or bone marrow diagnostic specimens was purified using the QIAcube Connect automated system with Qiagen AllPrep DNA/RNA/miRNA Universal Kits (80224). Purified RNA samples were then prepared for either strand specific polyadenylated enriched (polyA-enriched) messenger RNA libraries (*n* = 442) or strand specific ribosome RNA-depleted (rRNA-depleted) libraries (*n* = 1061) by the British Columbia Genome Sciences Center (BCGSC). Seventy-five base pair paired-end sequencing was performed on Illumina HiSeq 2000/2500 platforms. Sequence reads were aligned to the GRCh37 reference genome using BWA (v0.5.7)^56^. Reads were discarded based on mapping quality or if they failed the Illumina chastity filter and duplicate reads were marked using Picard (v1.11). Gene level coverage analysis was performed using the BCGSC pipeline v1.1 with Ensembl v69 annotations and were normalized based on RPKM (reads per kilobase per million mapped reads) or TPM (transcripts per million). Library preparation methodology (polyA-enriched versus rRNA-depleted) had less impact on median TPMs for LSC genes compared to the overall transcriptome (Supplementary Fig. 16).

### The Cancer Genome Atlas (TCGA) data processing

TCGA AML^57^ (LAML) RNA-sequencing data was downloaded from the Broad Institute GDAC Firehose repository. RPKM normalized gene level RNA-sequencing data were used in all LAML analyses.

### Leukemic stem cell 17 signature score

The LSC17 risk prediction model was described previously^11^. Briefly, RPKM normalized counts were log_2_-transformed after incrementing by 1. LSC17 scores for the TCGA LAML and our pediatric AML cohorts were calculated per patient as the sum of the log_2_-transformed RPKM expression values for the 17 genes weighted by the regression coefficients. High and low LSC17 groups were defined as above or below the median LSC17 score for the cohort, respectively.

### Survival analysis

Event free survival and overall survival analysis was performed using the Kaplan–Meier estimates. Event free survival (EFS) was defined as the time from enrollment to first event (relapse, induction failure, or death) or last follow-up. Overall survival (OS) was defined as the time from study enrollment to death or last follow-up. Cox proportional hazard regression models were employed to estimate hazard ratios (HR) for univariable and multivariable analyses of OS and EFS. Differences in OS and EFS between groups was determined using log-rank testing. All *p*-values are two-sided and without multiple-testing adjustments.

### Gene expression analysis

Principal component analysis (PCA) was performed using the prcomp R package, which is included R core base. PCA visualization was performed using the rgl (v0.100.54) R package. Nearest shrunken centroid analysis was performed using the pamr (v1.56.1) package^14^. Uniform manifold approximation and projection (UMAP) was performed using the umap (v0.2.6.0) R package. Gene set enrichment analysis (GSEA) was performed using command line tools^58^. Hierarchical clustering was performed using the made4 (v.1.58.0) R package^59^. Circos figures were generated using the Circos (v0.69-9) software package^60^.

### Signature training

Of the 48 enriched LSC genes identified by Ng et al.^11^, *FAM30A* (Entrez Gene ID 29064) and *KIAA0125* (Entrez Gene ID 9834) were merged into one Entrez Gene ID 9834 since they have since been found to be aliases of each another, resulting in 47 enriched LSC genes (LSC47). To extract a core subset genes from LSC47 that best explained patient outcomes in the training cohort, we used the same linear regression technique that formed the basis of LSC17 and is based on the LASSO (least absolute shrinkage and selection operator) algorithm as implemented in glmnet (v4.0-2) R package^61^, while enabling leave-one-out cross-validation to fit a Cox regression model as described previously^11^. TPM normalized counts were log_2_-transformed after incrementing by 1. A pediatric AML-specific LSC score was calculated per patient as the sum of the log_2_-transformed TPM expression values for the selected genes weighted by the regression coefficients. High and low LSC groups were defined as above or below the median LSC score for the cohort, respectively. The same analysis was also performed within each fusion training cohort to establish cytomolecular-specific regression coefficients and LSC scores. The coefficients for the entire cohort and cytomolecular subtypes were incorporated into a matrix of coefficients, where a column of coefficients is applied to a given patient based on underlying cytomolecular subtype to generate a pediatric LSC signature approach (LSC47) (Supplementary Table 1).

### Fusion calling

Fusion calls were made using CICERO (v1.8.1), STAR-Fusion (v.1.10.1), and Trans-ABySS (v.2.0.1)^62,63^. With respect to *CBFB-MYH11* breakpoints, overlapping calls between fusions callers were concordant at the level of exon transcript calling with each other. The remaining *CBFB-MYH11* fusions identified by RNA-sequencing were called by one or two of the three fusion callers.

### Statistics and reproducibility

Box plots were generated using ggplot2 (v.3.3.5)^64^. Box plots indicate the median and interquartile range, and whiskers indicate 1.5 times the interquartile range below and above the 25th and 75th percentile, respectively. *P*-values were calculated based on two-sided t-tests and the degree of significance is indicated by asterisks notation (**** <0.0001, *** ≥0.0001 and <0.001, ** ≥0.001 and <0.01, * ≥0.01 and <0.05). No statistical method was used to predetermine sample size for our analyses and no data were excluded from the analyses. Stratified randomization was performed based on fusion category to generate two cohorts for risk model training and validation.

### Disclaimer

The content is solely the responsibility of the authors and does not necessarily represent the official views of the National Institutes of Health.

### Reporting summary

Further information on research design is available in the Nature Research Reporting Summary linked to this article.

## Supplementary information

Supplementary

Reporting Summary

## Data Availability

The data generated for this study have been deposited in dbGaP under the dbGaP study ID phs000465.v21.p8 and in the TARGET Data Matrix at the TARGET Data Coordinating Center [https://target.nci.nih.gov/dataMatrix/TARGET_DataMatrix.html]. NIH TARGET genomic sequencing data is available through controlled-access as part of the NIH Genomic Data Sharing Policy to ensure that all approved investigators and institutions abide by the NIH Genomic Data User Code of Conduct, the terms of the Data Use Certification, and the Security Best Practices for Controlled Access Data (for more details, https://grants.nih.gov/grants/guide/notice-files/NOT-OD-14-124.html. Data access is restricted for academic use and can be requested here (https://www.ncbi.nlm.nih.gov/projects/gap/cgi-bin/study.cgi?study_id=phs000465.v21.p8). Based on our own experiences and if approved, data access is typically granted within a week of request. TCGA AML (LAML) RNA-sequencing is available for download through the Broad Institute GDAC Firehose repository [https://gdac.broadinstitute.org/]. The GRCh37 reference genome is available for download through the Michael Smith Genome Science Center [https://www.bcgsc.ca/downloads/genomes/9606/hg19]. Source data are provided with this paper.

## Source data

Source Data
