# Supplementary material for: Integrated stem cell signature and cytomolecular risk determination in pediatric acute myeloid leukemia
Source: Nat Commun. 2022 Sep 19;13:5487. doi: 10.1038/s41467-022-33244-6 (PMC9485122; doi:10.1038/s41467-022-33244-6)
Supplement: Supplementary file 1 — Supplementary [file 41467_2022_33244_MOESM1_ESM.pdf]

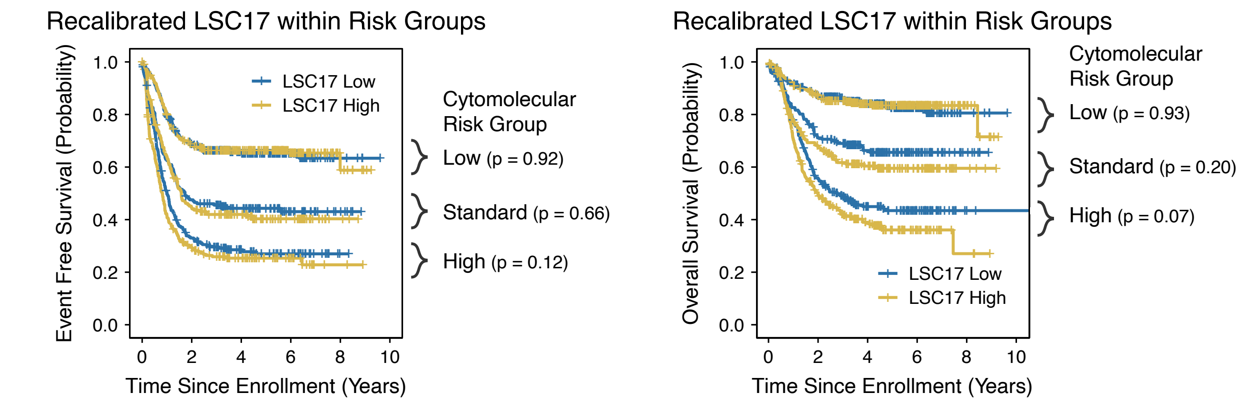

No. of patients at risk

LSC17 Low	290	188	124	44	5	0
LSC17 High	290	188	129	64	9	0
LSC17 Low	204	95	62	25	7	0
LSC17 High	205	87	58	26	3	0
LSC17 Low	257	81	39	21	1	0
LSC17 High	257	70	40	14	2	0

No. of patients at risk

LSC17 Low	290	237	158	55	5	0
LSC17 High	290	239	166	78	10	0
LSC17 Low	204	139	93	37	7	0
LSC17 High	205	130	79	37	4	0
LSC17 Low	257	136	66	32	2	1
LSC17 High	257	115	63	19	3	0

Supplementary Figure 1: Kaplan-Meier estimates for the probability of event free survival and overall survival among patients stratified based on cytomolecular status. In contrast to the corresponding main figures, LSC17 low versus high assignments were re-classified within each cytomolecular strata, such that the median within a given cohort represents the new breakpoint between LSC17 low versus high. Despite re-classifying LSC17 status, LSC17 scores are not predictive within cytomolecular risk groups. Survival differences were determined using the log-rank test (two-sided and without multiple-testing adjustments).

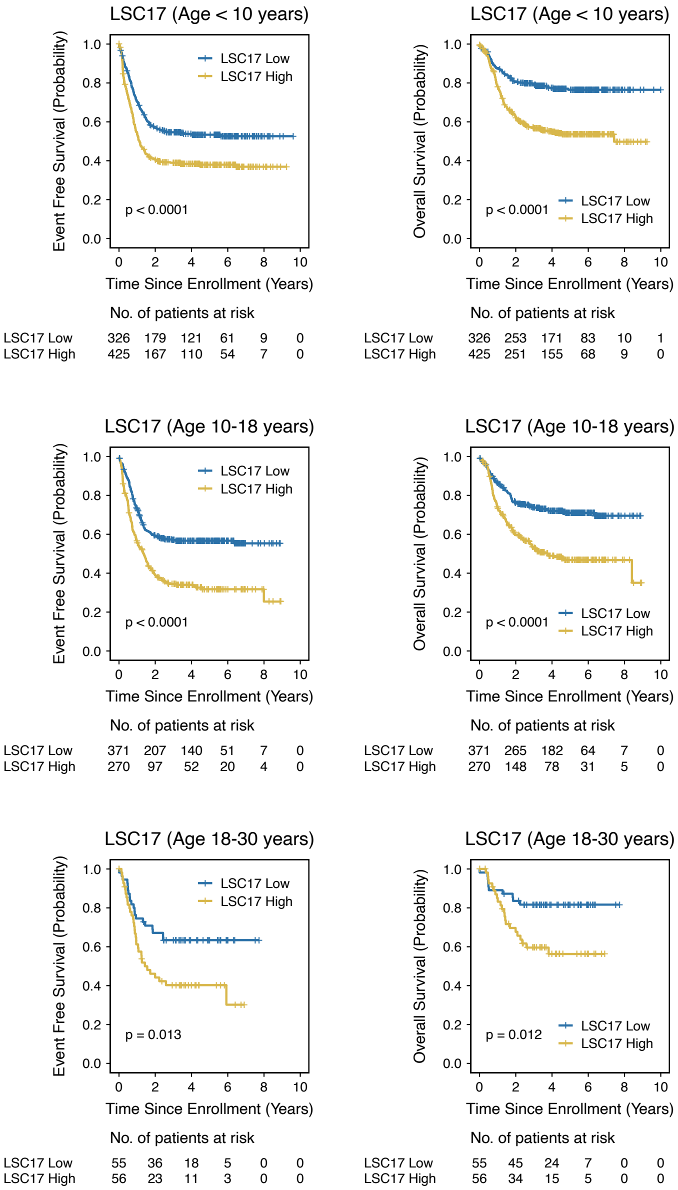

Supplementary Figure 2: Kaplan-Meier estimates for the probability of event free survival (left) and overall survival (right) based on LSC17 scores and stratifying patients based on age. Children (ages 0-10 years), adolescents (ages 10-18 years), and young adults (ages 18-30 years). Survival differences were determined using the log-rank test (two-sided and without multiple-testing adjustments).

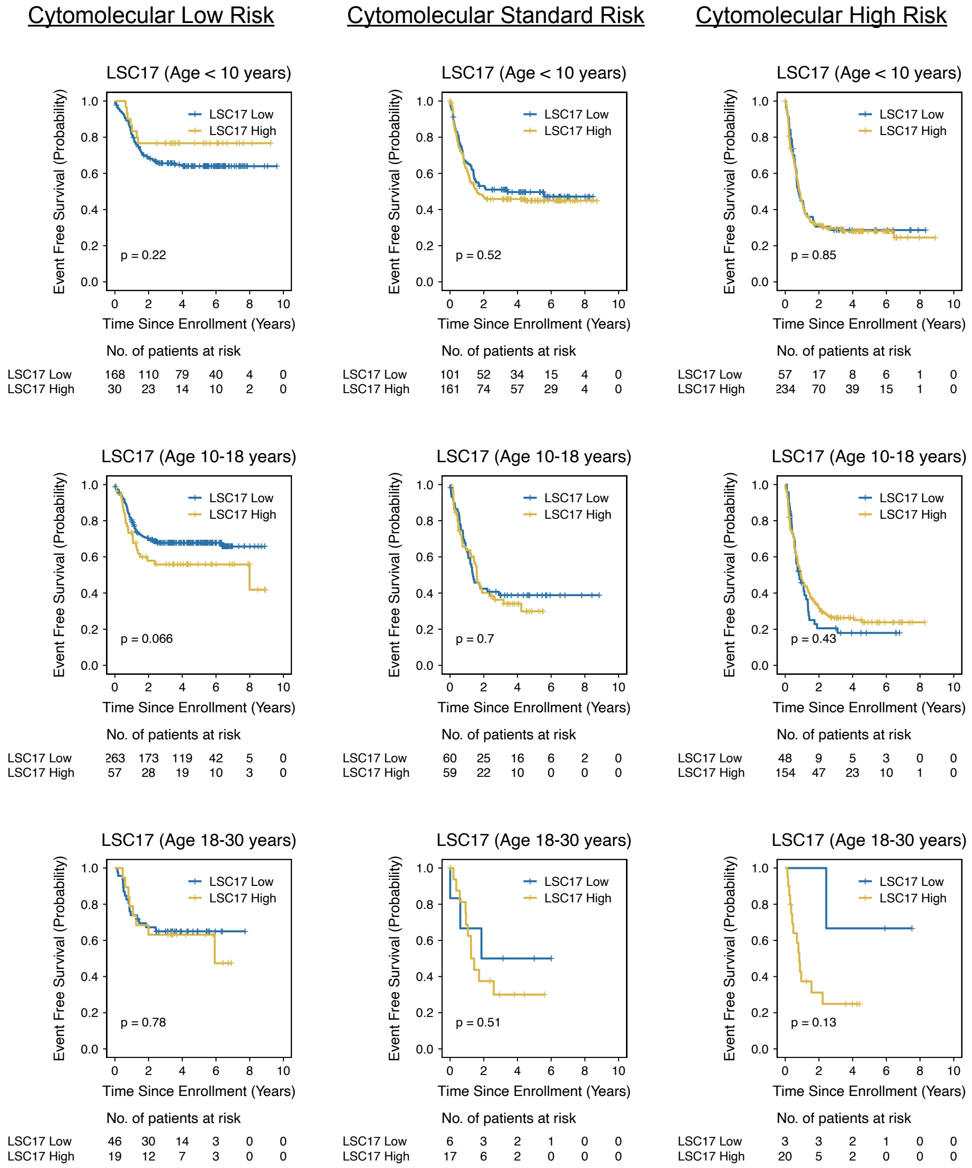

Supplementary Figure 3: Kaplan-Meier estimates for the probability of event free survival based on LSC17 scores and stratifying patients based on age (as described in Supplementary Fig. 2) and cytomolecular risk groups. Survival differences were determined using the log-rank test (two-sided and without multiple-testing adjustments).

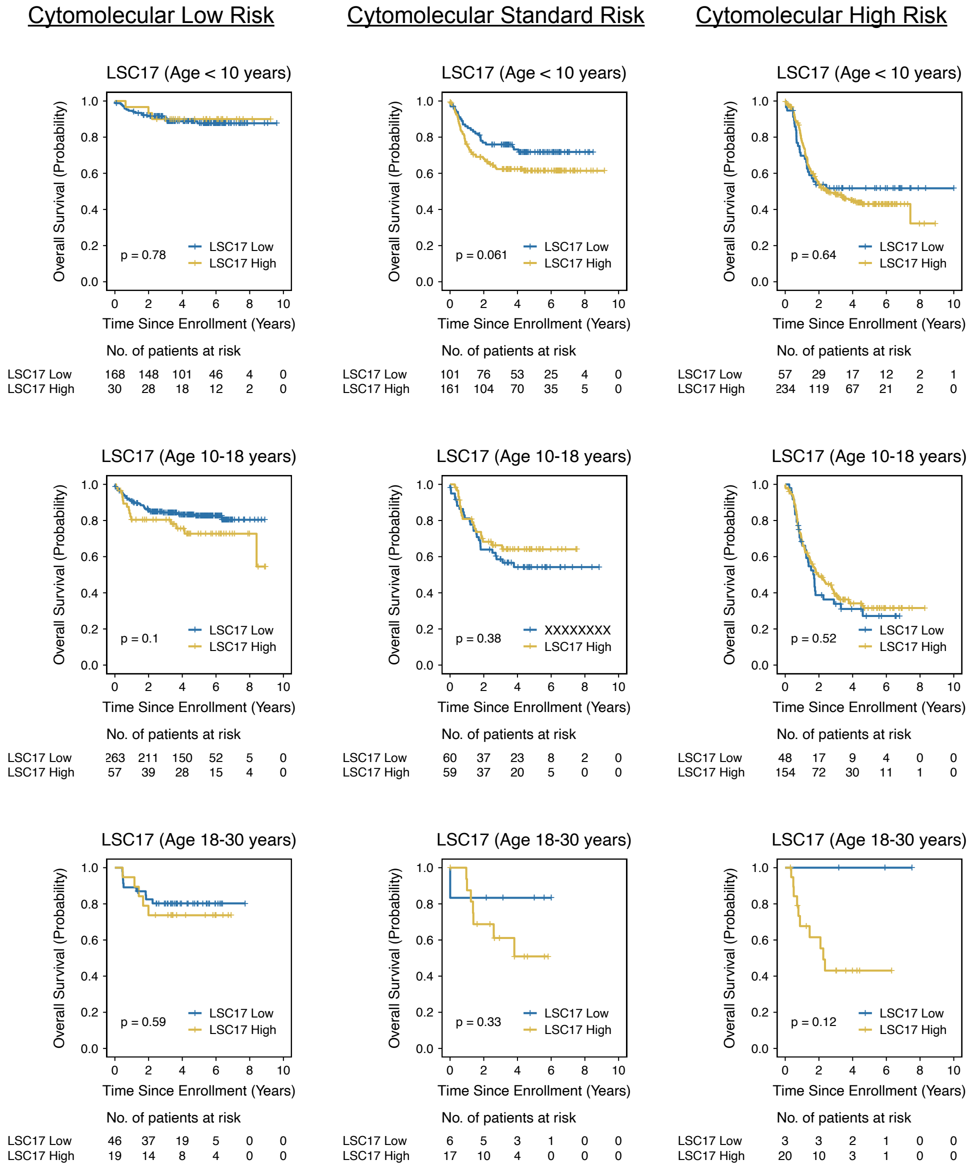

Supplementary Figure 4: Kaplan-Meier estimates for the probability of overall survival based on LSC17 scores and stratifying patients based on age (as described in Supplementary Fig. 2) and cytomolecular risk groups. Survival differences were determined using the log-rank test (two-sided and without multiple-testing adjustments).

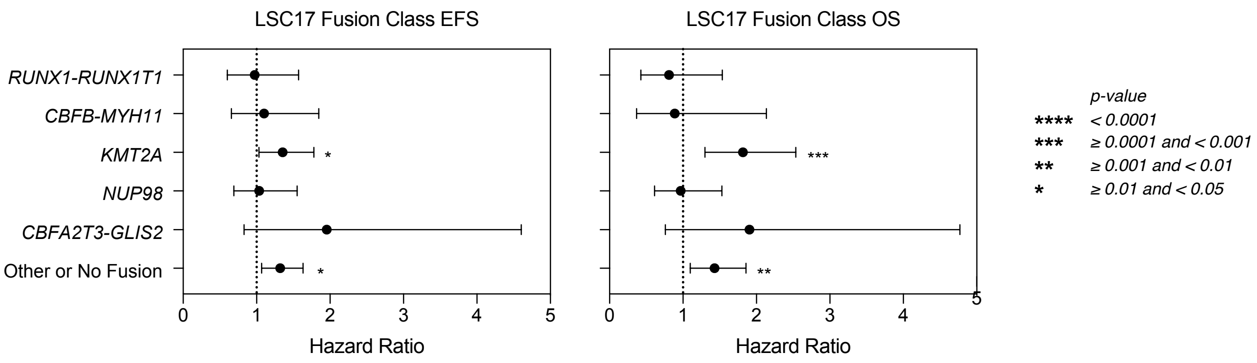

Supplementary Figure 5: Hazard ratios with 95% confidence intervals for EFS and OS as a function of LSC17 risk group (high versus low) across fusion classes. Among *KMT2A* or OTHER AMLs, LSC17 scores significantly predicted favorable outcomes. Conversely, patients diagnosed with AMLs containing other fusions (*RUNX1-RUNX1T1*, *CBFB-MYH11*, *NUP98*, or *CBFA2T3-GLIS2*) had similar outcomes regardless of LSC17 score. Since the *CBFA2T3-GLIS2* fusion class only contains one patient assigned to a LSC17 low score, these results were generated after reassigning LSC17 category (low versus high) based on median score within a given fusion class. Results are similar regardless of whether LSC17 is recalibrated or not (n = 1503 patients). Survival differences were determined using the log-rank test (two-sided and without multiple-testing adjustments).

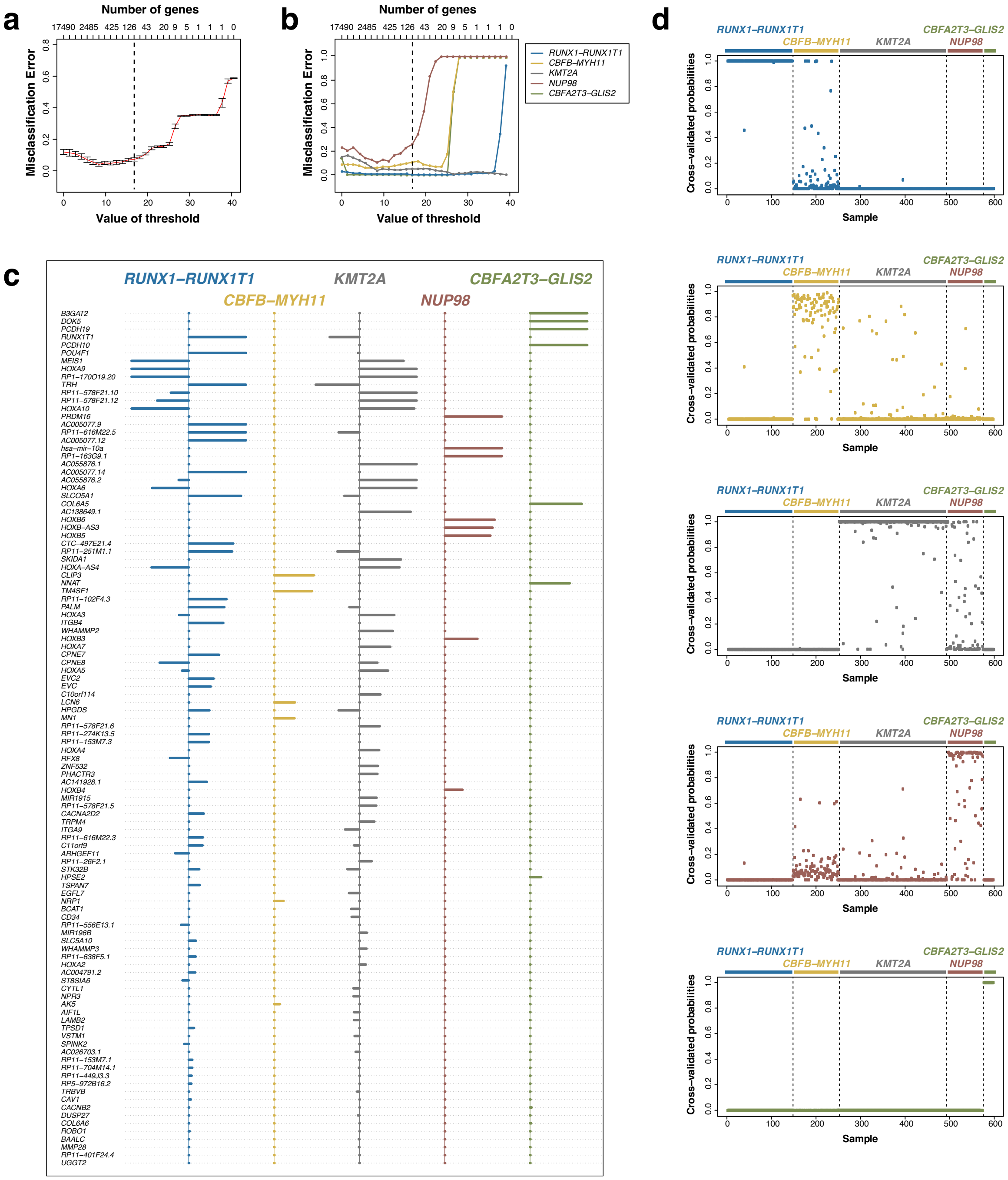

Supplementary Figure 6: The nearest shrunken centroids approach allows for cancer class prediction from gene expression profiling. This approach was applied to the classification of AMLs based on underlying driver fusion to rank order genes based on predictive power. (a) The misclassification error rate with 95% confidence intervals is shown as a function of number of selected genes. (b) The amount of shrinkage threshold value = 18 is chosen and yields a subset of 104 selected genes to form a fusion prediction model. (c) 104 gene set representing the shrunken centroids principal components. (d) Using the selected 104 gene set prediction model, the estimated probability for correctly predicting associated fusion class is robust (n = 1061 patients from ribosomal RNA depletion RNA-seq cohort).

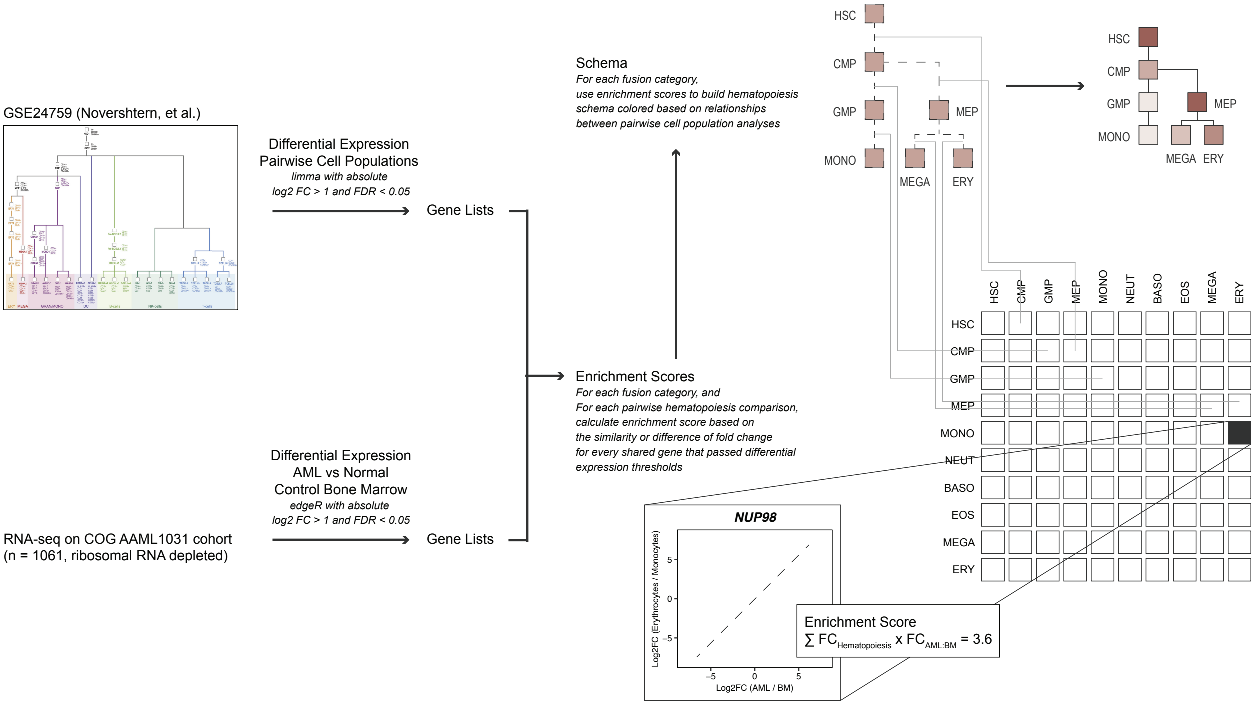

Supplementary Figure 7: To correlate leukemia transcriptional landscape with hematopoietic and myeloid progenitor differentiation transcriptional states, differential expression analysis was performed using edgeR with absolute log2 fold change threshold of greater than 1 and false-discovery rate (FDR) threshold of less than 0.05. Similarly, differential expression analysis was performed on every pairwise comparison within a previously described hematopoiesis cell population gene expression study (Novershtern, *et al.*) (e.g., HSC versus CMP, HSC versus GMP, etc.) using limma with the same log2 fold change and FDR thresholds as above. Enrichment scores for a given fusion category (*RUNX1-RUNX1T1*, *CBFB-MYH11*, *KMT2A*, *NUP98*, or *CBFA2T3-GLIS2*) were calculated for each cell population by plotting log2 fold change versus log2 fold change for the two differential expression cohorts, and summing the product of the two-fold changes with one another, such that positive enrichment scores indicate that (for example) *NUP98* AMLs are enriched in an "Erythrocytes versus Monocytes" gene set.

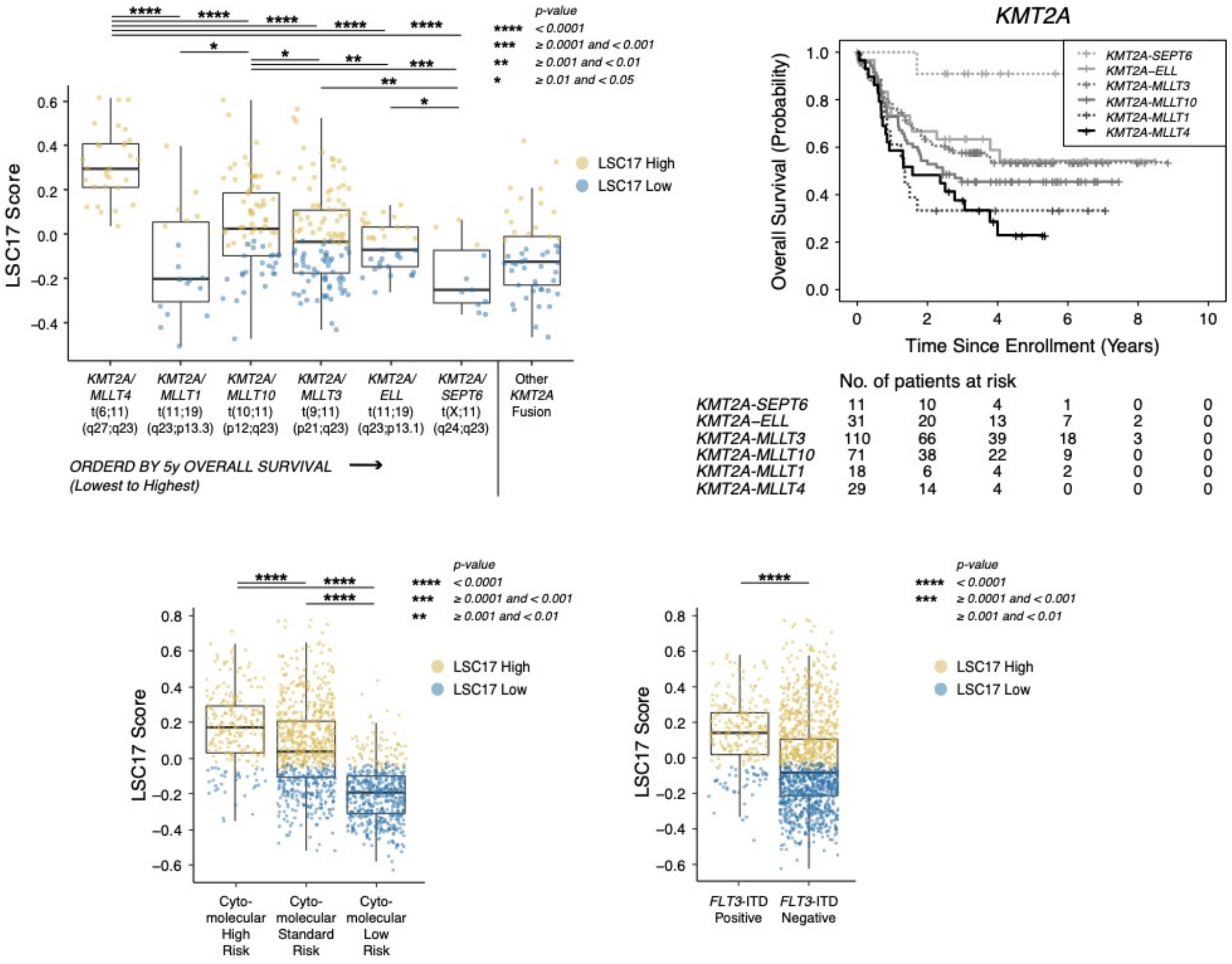

Supplementary Figure 8: Box plot of LSC17 scores categorized based on *KMT2A* fusion gene partner reveal that LSC17 scores also significantly correlate with fusion partner, as well as overall survival (n = 315 patients from the *KMT2A* cohort) (top panels). Similar findings are observed when AMLs are grouped based on cytomolecular risk group (n = 1503 patients) and *FLT3*-ITD status (n = 293 patients).

Survival differences were determined using the log-rank test (two-sided and without multiple-testing adjustments). Box plot data are presented as median values with hinges corresponding to the 25th or 75th percentiles and whiskers corresponding to 1.5 times the inter-quartile range. Box plot p-values were calculated based on two-sided t-tests. Source data are provided as a Source Data file.

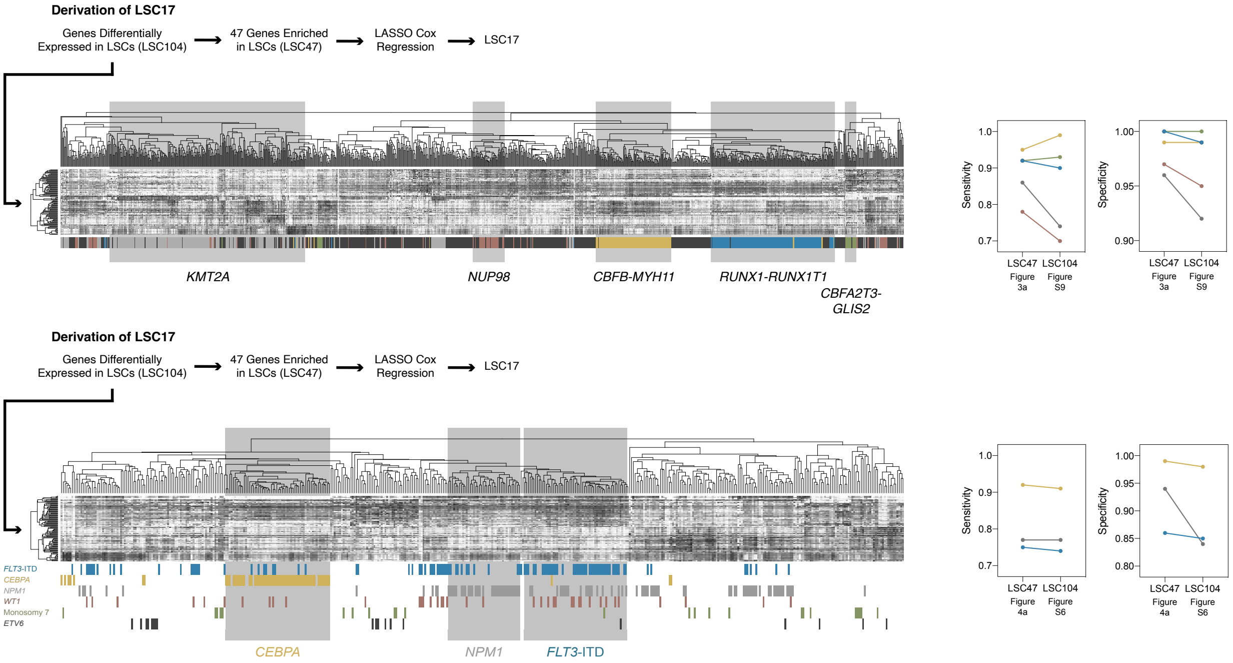

Supplementary Figure 9: The LSC17 gene signature was previously generated based on LASSO Cox regression on 47 genes enriched in LSC AML cell populations (LSC47). There were additional genes that were enriched in the LSC negative populations, resulting in a 104 gene set (LSC104) of differentially expressed genes between LSC+ and LSC– populations. Performing hierarchical clustering based on LSC47 versus LSC104 (Fig. 3a and 4a versus above supplementary heatmap figures, respectively) reveals that LSC104 does not contribute additional ability to discriminate fusion classes from one another and in fact is associated with diminished sensitivity/specificity in this regard (side panel).

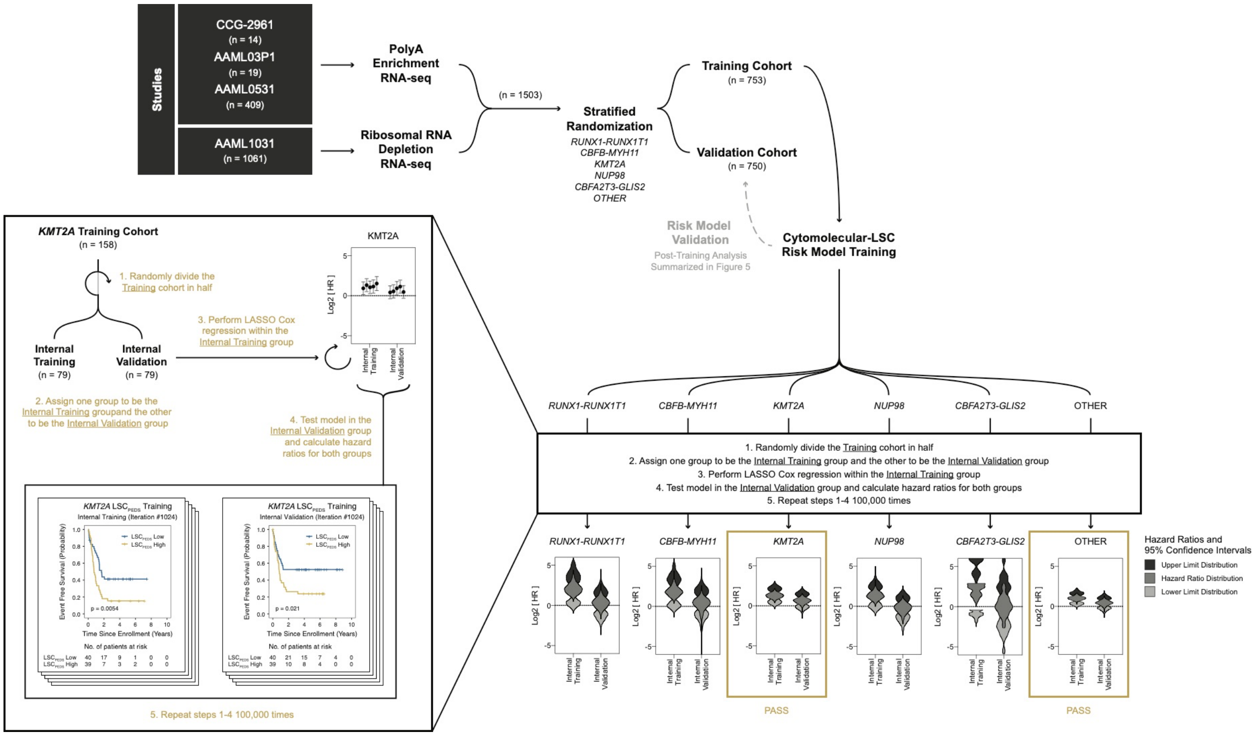

Supplementary Figure 10: To perform internal cross validation analysis, training cohort fusion subgroups were iteratively and randomly divided in half. LASSO Cox regression modeling analysis was performed on one half to determine whether iterative gene signatures remain predictive in the non-modeled half. This iterative process was repeated 10,000 times and hazard ratios (and their associated 95% confidence intervals) for each iteration were plotted. Violin plots represent the distribution of these iterations.

RUNX1 transcriptional signature “mediator genes” (Hornung, et al.)

CD109, HOPX, KIAAA0125, GPR56*, NGFRAP1**
* Included in LSC47

CBFB-MYH11 fusion breakpoint (Schwind, et al.)

CBFB exon 5 and MYH11 exon 33 (type A fusion) versus non-type A fusions

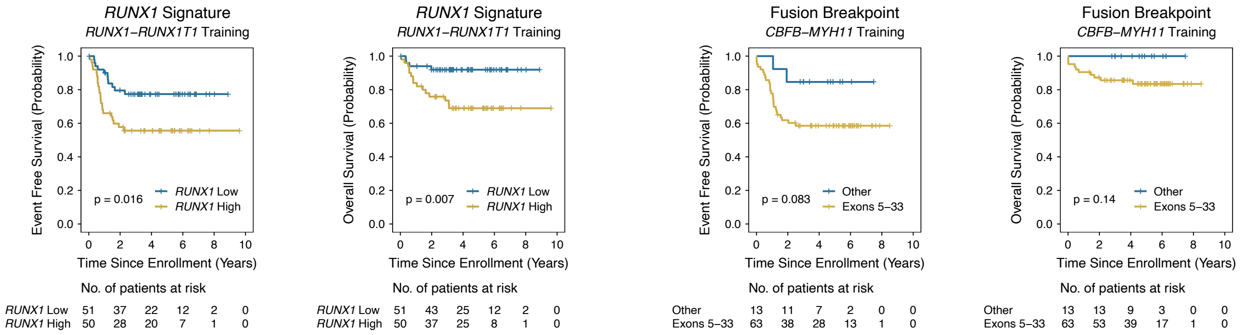

Supplementary Figure 11: Kaplan-Meier estimates for the probability of EFS and OS within the training cohort based on a RUNX1 transcriptional signature (left panel). Kaplan-Meier estimates for the probability of EFS and OS within the training cohort for CBFB-MYH11 AMLs based on fusion breakpoint. AMLs with CBFB exon 5 and MYH11 exon 33 fusions have significantly worse EFS outcomes compared to other breakpoints (right panel). Survival differences were determined using the log-rank test (two-sided and without multiple-testing adjustments).

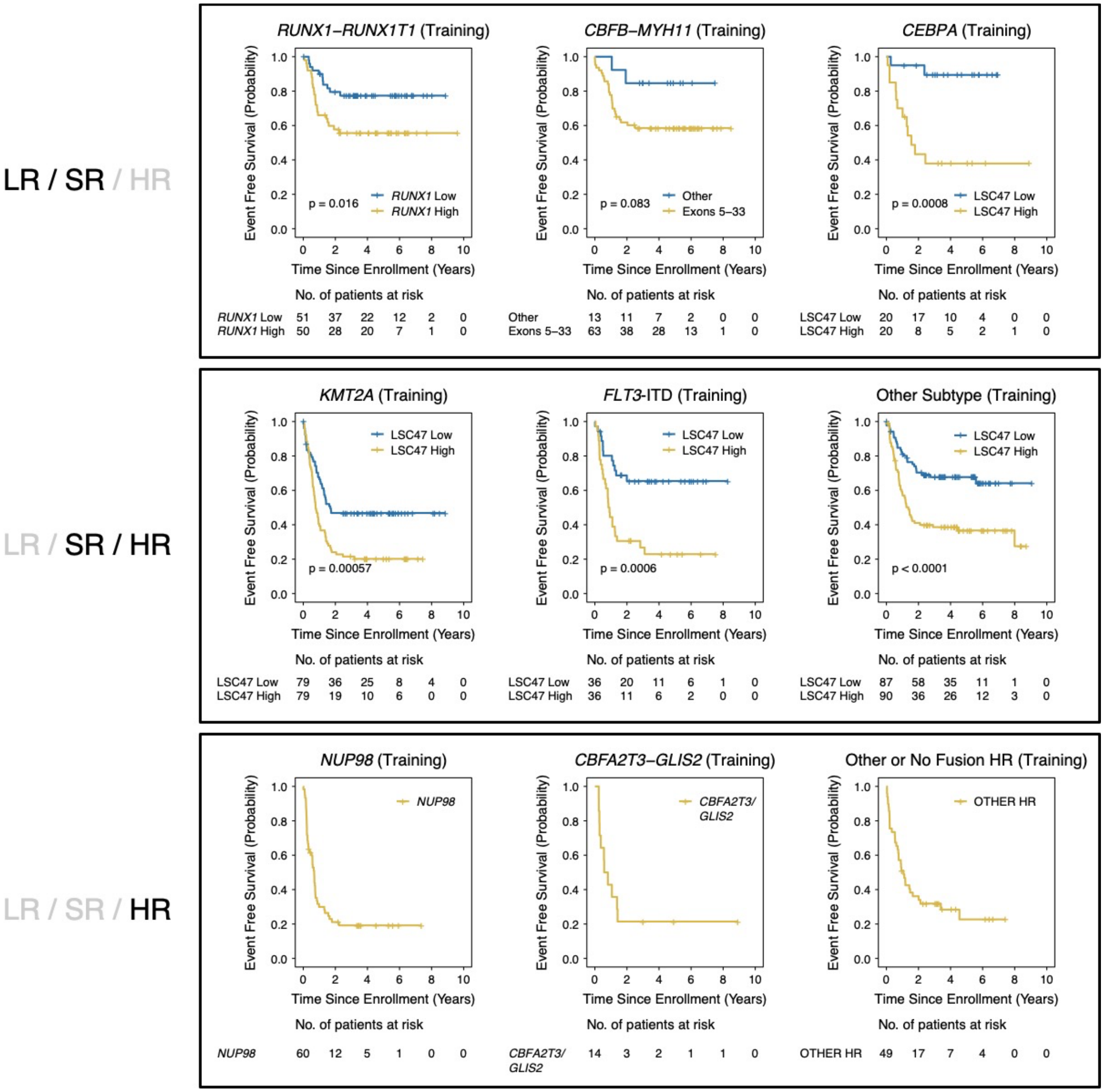

Supplementary Figure 12: Kaplan-Meier estimates for the probability of EFS within the training cohort based on cytomolecular category with proposed risk criteria applied. *RUNX1* transcriptional signature is based on Hornung, *et al.* Exons 5-33 refers to *CBFB-MYH11* transcripts with *CBFB* exon 5 and *MYH11* exon 33 breakpoints. *CEBPA*, *FLT3*-ITD, and Other Subtype AMLs are risk stratified based on LSC47. *NUP98* and *CBFA2T3-GLIS2* AMLs were labeled as "high-risk" due to uniformly unfavorable outcomes. Other cytomolecular subtypes that are consistently associated with unfavorable outcomes were filtered and labeled as "high-risk" prior to any subsequent analysis and include AMLs with monosomy 7, deletion 5q, *MLLT10* rearrangements (non-*KMT2A*), or *ETV6* rearrangements or deletions. Survival differences were determined using the log-rank test (two-sided and without multiple-testing adjustments).

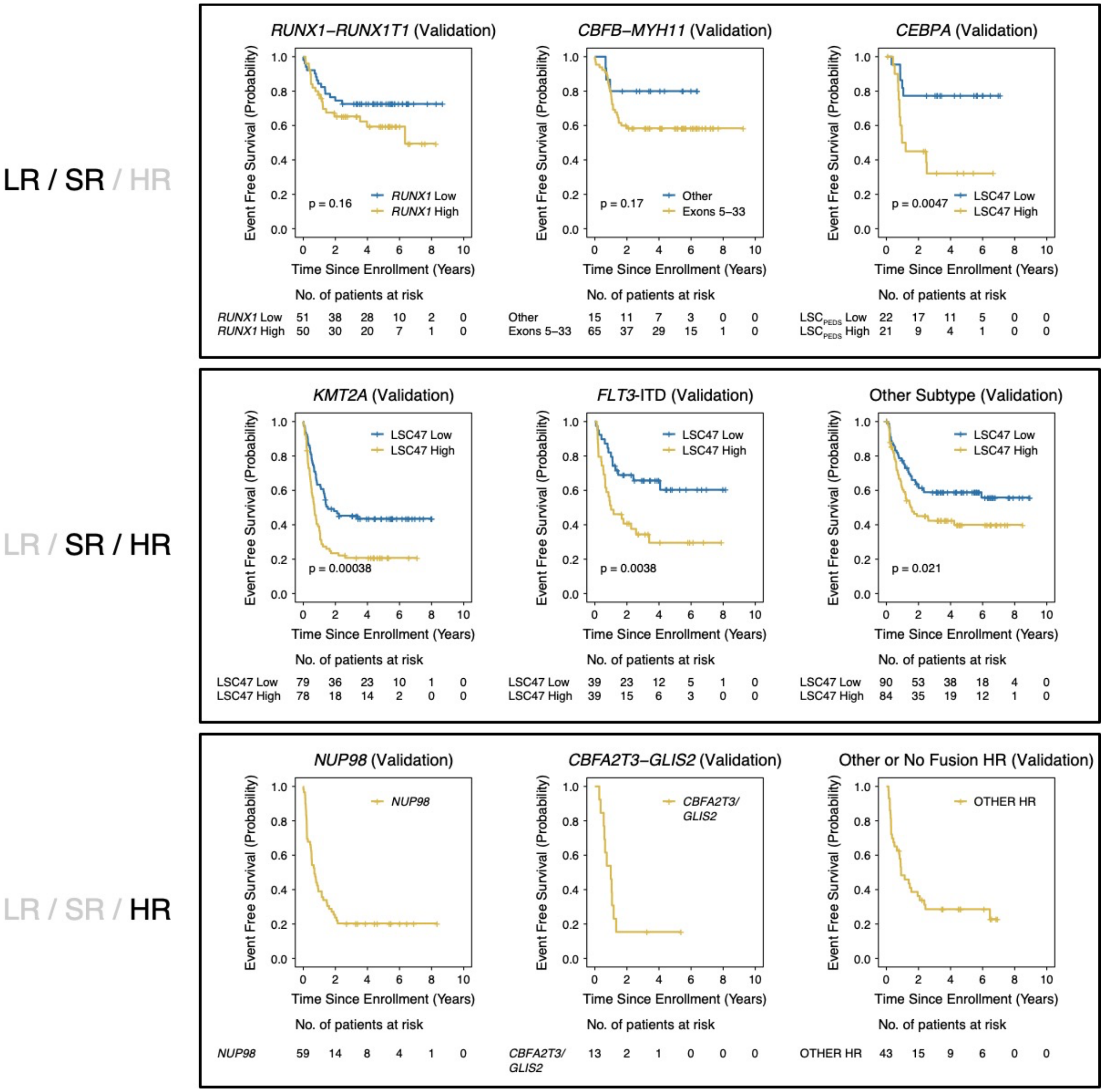

Supplementary Figure 13: Kaplan-Meier estimates for the probability of EFS within the validation cohort based on cytomolecular category with proposed risk criteria applied. For further details, refer to Supplementary Fig. 12. Survival differences were determined using the log-rank test (two-sided and without multiple-testing adjustments).

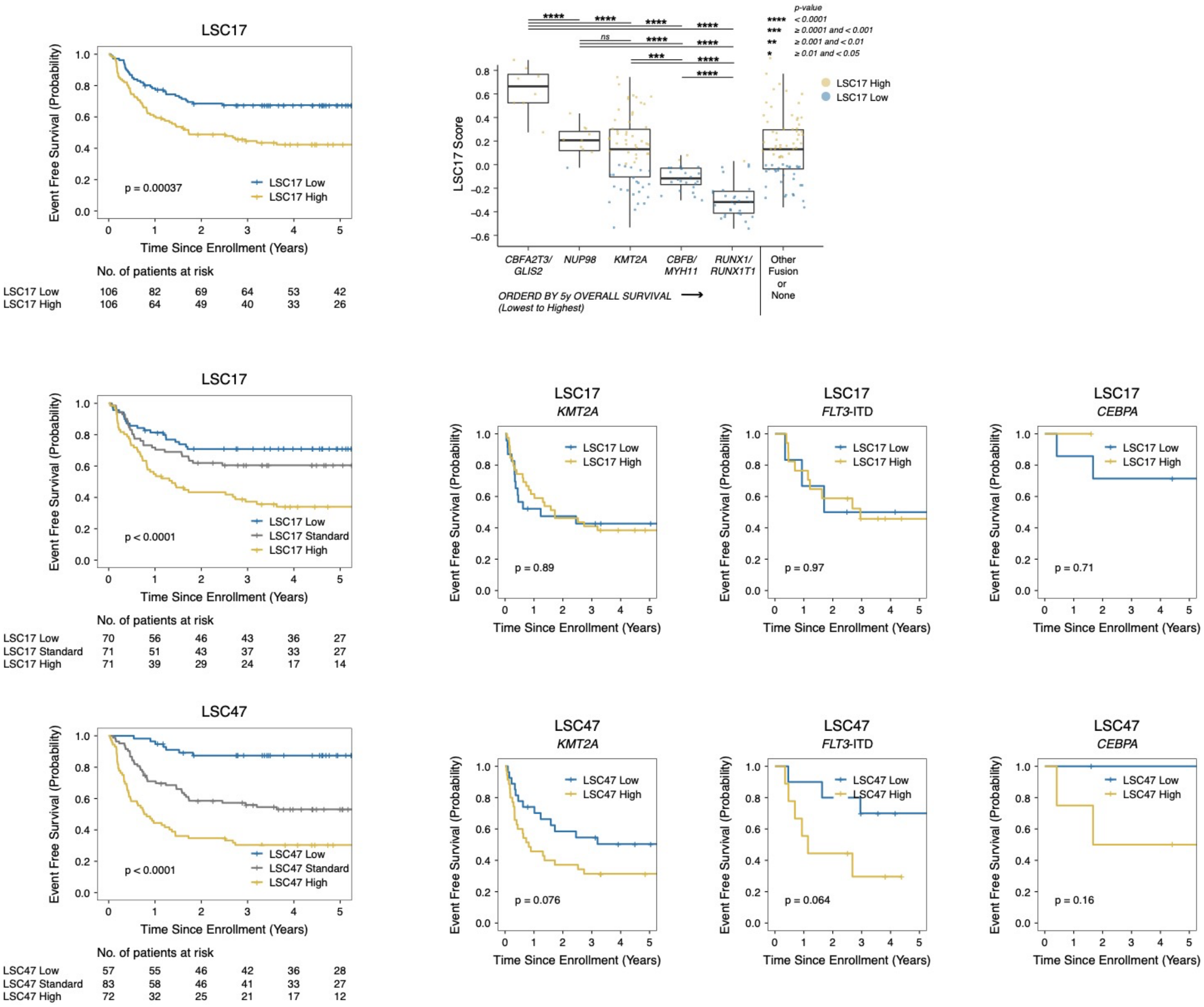

Supplementary Figure 14: St. Jude AML08 independent validation cohort. Kaplan-Meier estimates for the probability of EFS stratified based on LSC17 scores (top left panel). Box plot of LSC17 scores categorized based on fusion status (n = 230) (top right panel). Kaplan-Meier estimates for the probability of EFS based on LSC17 versus LSC47 (middle and bottom panels, respectively).

Survival differences were determined using the log-rank test (two-sided and without multiple-testing adjustments). Box plot data are presented as median values with hinges corresponding to the 25th or 75th percentiles and whiskers corresponding to 1.5 times the inter-quartile range. Box plot p-values were calculated based on two-sided t-tests. Source data are provided as a Source Data file.

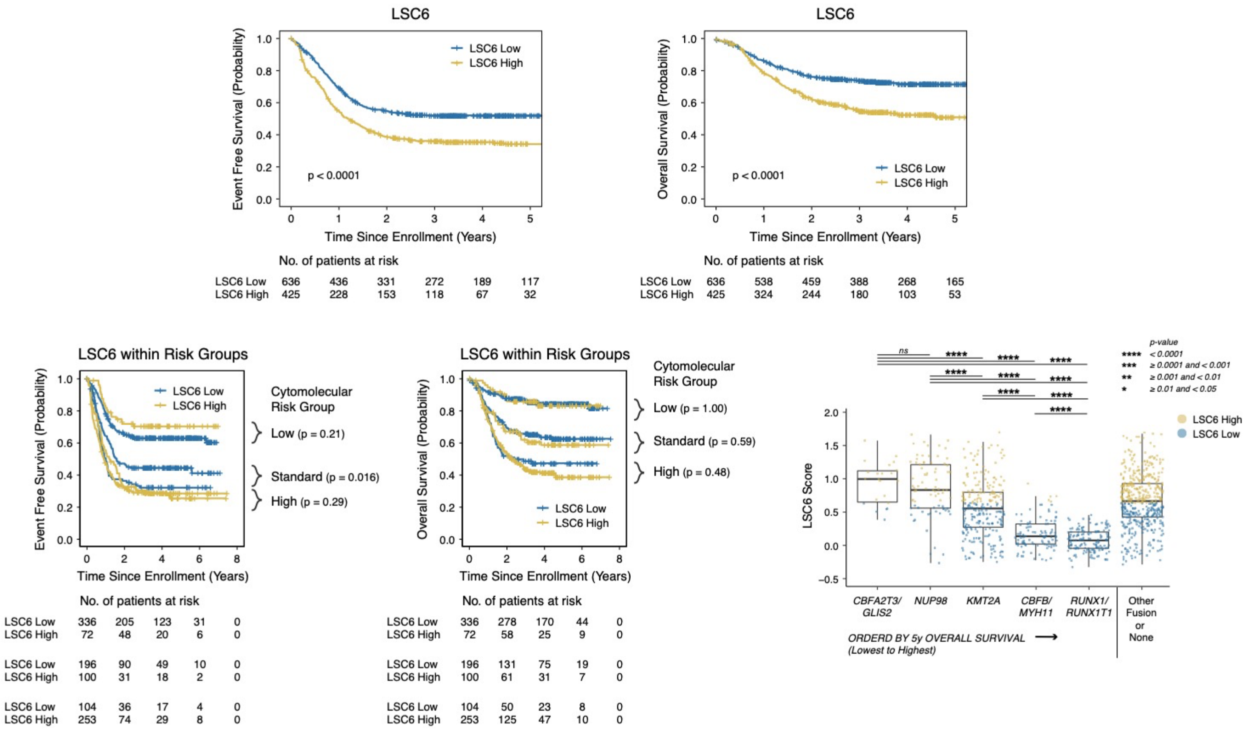

Supplementary Figure 15: Evaluation of LSC6. Kaplan-Meier estimates for the probability of EFS and OS based on LSC6 (top panel). Kaplan-Meier estimates for the probability of EFS and OS based on LSC6, but also stratified based on cytomolecular risk (bottom left panel). Box plot of LSC6 scores categorized based on fusion status (n = 1061 patients from ribosomal RNA depletion RNA-seq cohort) (bottom right panel).

Survival differences were determined using the log-rank test (two-sided and without multiple-testing adjustments). Box plot data are presented as median values with hinges corresponding to the 25th or 75th percentiles and whiskers corresponding to 1.5 times the inter-quartile range. Box plot p-values were calculated based on two-sided t-tests. Source data are provided as a Source Data file.

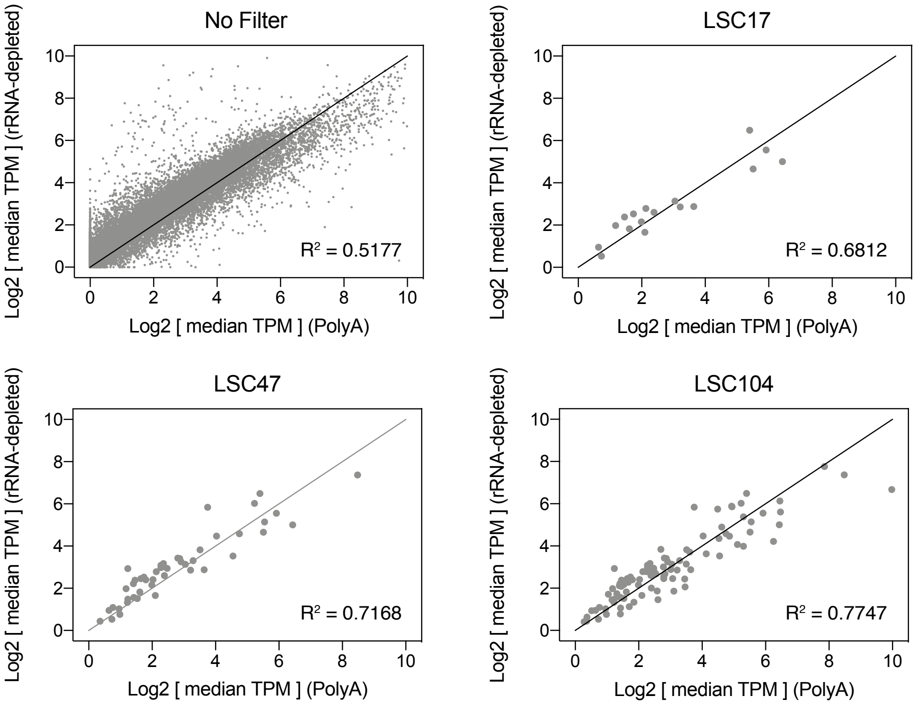

Supplementary Figure 16: Gene expression values ($\log_2$ transformed median TPM) compared based on library preparation methodology (rRNA-depleted versus polyA-enriched). The coefficients of determination are higher when focusing on comparisons that include LSC genes only.

**Supplementary Table 1.** LSC PEDS										
Gene	LSC17	LSC PEDS NO FILTER	LSC PEDS KMT2A	LSC PEDS CEBPA	LSC PEDS FLT3-ITD	LSC PEDS OTHER	Ng, et al. Alias	Other Aliases	Ng, et al. Entrez ID	Updated Entrez ID
CD34	0.03380	0.04618	0.01258	-0.00479	0.02804	0.03100			947	
SPINK2	0	0.04217	0	-0.00849	0.03782	0			6691	
LAPTM4B	0.00582	0.01152	0	0.00795	-0.00024	-0.00248			55353	
HOXA5	0	0.00418	0.01167	0	-0.01093	-0.00824			3202	
GUCY1A3	0	0.00744	0.00562	0.00651	0	0			2982	
SHANK3	0	0.01449	0	0.00620	-0.01239	0.00655			85358	
ANGPT1	0	0.00354	0.00561	0	0	0			284	
ARHGAP22	-0.01380	0	0	0.00697	0	0.00074			58504	
SMIM24	-0.02260	-0.00191	-0.00083	0	-0.00161	0	*LOC284422*	*C19orf77*	284422	
MYCN	0	0.00050	0	-0.01505	0.00000	0.01461			4613	
MAMDC2	0	-0.01386	0	0.02170	0.01849	0.01152			256691	
PRSS57	0	-0.02234	-0.01274	0	0.01580	-0.01841	*PRSSL1*		400668	
KIAA0125/FAM30A	0.01960	0	0	0	0	0			9834/29064	9834
GPSM1	0	-0.00924	0	0.02509	-0.01925	-0.00804			26086	
HOXA9	0	0.02564	0	0	-0.01139	-0.01034			3205	
MMRN1	0.02580	0.03547	0.01515	-0.01162	-0.00260	-0.00282			22915	
FSCN1	0	0	0.00770	-0.04277	0.00159	-0.01671			6624	
DNMT3B	0.08740	0.01563	0	-0.01223	-0.00547	0			1789	
HOXA6	0	0.01803	0.01196	0	-0.01849	-0.01248			3203	
AIF1L	0	0	0	0	0.02361	0.03458			83543	
SOCS2	0.02710	0.02196	0.00940	0.02650	-0.01891	0			8835	
CDK6	-0.07040	-0.01046	0	0.00323	-0.01186	0			1021	
FAM69B	0	-0.04990	0	-0.03190	0	-0.01319		*DIPK1B*	138311	
NGFRAP1	0.04650	0	0	-0.00783	-0.01711	-0.00199		*BEX3*	27018	
FAM212A	0	0.00364	0	-0.03392	0	-0.00124	*C3orf54*	*INKA1*	389119	
CPXM1	-0.02580	-0.03178	-0.00068	0	0	-0.02835			56265	
TNFRSF4	0	0.01985	0	0.02516	0.03843	0.03248			7293	
ZBTB46	-0.03470	0	0	-0.01421	0	0			140685	
DPYSL3	0.02840	-0.00442	0.00363	0.00552	0	0			1809	
NYNRIN	0.00865	0.02902	0	-0.02660	0	0.01726			57523	
COL24A1	0	0	-0.00067	0.00041	-0.01355	-0.00986			255631	
SKIDA1	0	0	0	-0.00359	-0.01696	-0.01769	*C10orf140*		387640	
SPNS2	0	0	0	0	-0.01744	0			124976	
GPR56	0.05010	0.02054	0	0	0.00285	0.04001		*ADGRG1*	9289	
AKR1C3	-0.04020	0	0	-0.00921	0.00021	-0.01534			8644	
FLT3	0	-0.02050	0	-0.00224	0	-0.00504			2322	
TFPI	0	-0.02446	0	0.01926	-0.01026	-0.00324			7035	
KCNK17	0	-0.03478	-0.00673	-0.03866	-0.00238	-0.00215			89822	
EPDR1	0	0.00033	0.00303	0	0	-0.01727			54749	
GCSAML	0	0	0	0.03156	-0.00211	0	*C1orf150*		148823	
BIVM	0	0	0	-0.02460	0	-0.00206			54841	
H2AFY2	0	0	0	-0.01311	-0.01444	0		*MACROH2A2*	55506	
VWF	0	0.00086	0	0	0.01880	0.02591			7450	
EMP1	0.01460	0	0.00479	0	0.01824	0.00790			2012	
AGER	0	0	0	-0.02706	0.00020	0	*RAGE*	*MOK*	5891	
ATP8B4	0	-0.01235	0	0.02649	-0.00563	0			79895	
GATA2	0	-0.00403	0	0	0	-0.00336			2624	

Supplementary Table 2. Univariable Survival Analysis of the Validation Cohort

	KMT2A fusion (n = 156)§		*FLT3*-ITD (n = 77)§		*CEBPA* mutation (n = 43)§		Other Subtype (n = 156)§	
Event Free Survival Covariate	Hazard Ratio (95% CI)†	P-value‡	Hazard Ratio (95% CI)†	P-value‡	Hazard Ratio (95% CI)†	P-value‡	Hazard Ratio (95% CI)†	P-value‡
Age	1.03 (0.99-1.06)	0.099	0.99 (0.93-1.06)	0.871	1.08 (0.96-1.22)	0.217	1.00 (0.97-1.03)	0.958
WBC count ≥ 50,000/μL	1.28 (0.87-1.89)	0.215	1.95 (1.04-3.66)	0.038	2.09 (0.78-5.59)	0.142	1.67 (1.00-2.78)	0.049
FLT3-ITD mutation*	0.59 (0.19-1.88)	0.375	NA	NA	0.44 (0.10-1.93)	0.278	NA	NA
NPM1 mutation*	NA	NA	0.42 (0.18-1.01)	0.053	NA	NA	NA	NA
CEBPA mutation*	NA	NA	NA	NA	NA	NA	NA	NA
LSC17 score	3.66 (1.57-8.56)	0.003	2.57 (0.51-13.0)	0.254	0.33 (0.01-16.0)	0.578	2.04 (0.73-5.68)	0.173
LSC47 score	4.96 (2.22-11.1)	<0.001	26.6 (4.76-148.3)	<0.001	8.78 (1.27-60.9)	0.028	5.02 (1.54-16.3)	0.007
HR fusion gene partner**	1.69 (1.15-2.49)	0.008	NA	NA	NA	NA	NA	NA

Abbreviations: WBC, white blood cells; ITD, internal tandem duplication; LSC, leukemia stem cell; HR, high risk

§ Number of patients with full clinical annotation within the validation cohort
† 95% confidence interval
‡ P-value calculated using the two-sided Wald test
* NA = not applicable due to absence, category defining, or filter defining (i.e., Other Subtype) mutation(s)
** NA = not applicable due to solitary fusion gene partner

Supplementary Table 3. Multivariable Survival Analysis

KMT2A (n = 156)§ EFS Covariate	Hazard Ratio (95% CI)†	P-value‡
LSC17 score	1.40 (0.45-4.41)	0.561
LSC47 score	3.26 (0.95-11.2)	0.059
HR fusion gene partner	1.19 (0.75-1.90)	0.455

FLT3-ITD (n = 77)§ EFS Covariate	Hazard Ratio (95% CI)†	P-value‡
WBC count ≥ 50,000/µL	1.90 (1.01-3.56)	0.047
LSC47 score	26.8 (4.71-153.0)	<0.001

CEBPA mutation (n = 43)§ EFS Covariate	Hazard Ratio (95% CI)†	P-value‡
LSC47 score only significant univariable covariate		

Other Subtype (n = 156)§ EFS Covariate	Hazard Ratio (95% CI)†	P-value‡
WBC count ≥ 50,000/µL	1.65 (0.99-2.75)	0.055
LSC47 score	4.99 (1.52-16.4)	0.008

Abbreviations: WBC, white blood cells; ITD, internal tandem duplication; LSC, leukemia stem cell; EFS, event free survival; HR, high risk

§ Number of patients with full clinical annotation within the validation cohort

† 95% confidence interval

‡ P-value calculated using the two-sided Wald test
