# Supplementary material for: Integrated stem cell signature and cytomolecular risk determination in pediatric acute myeloid leukemia
Source: Nat Commun. 2022 Sep 19;13:5487. doi: 10.1038/s41467-022-33244-6 (PMC9485122; doi:10.1038/s41467-022-33244-6)
Supplement: Supplementary file 2 — Reporting Summary [file 41467_2022_33244_MOESM2_ESM.pdf]

nature portfolio

Corresponding author(s): Benjamin J. Huang, MD

Last updated by author(s): Aug 19, 2022

Reporting Summary

Nature Portfolio wishes to improve the reproducibility of the work that we publish. This form provides structure for consistency and transparency in reporting. For further information on Nature Portfolio policies, see our Editorial Policies and the Editorial Policy Checklist.

Statistics

For all statistical analyses, confirm that the following items are present in the figure legend, table legend, main text, or Methods section.

n/a	Confirmed	
☐	☒	The exact sample size (*n*) for each experimental group/condition, given as a discrete number and unit of measurement
☐	☒	A statement on whether measurements were taken from distinct samples or whether the same sample was measured repeatedly
☐	☒	The statistical test(s) used AND whether they are one- or two-sided<br>*Only common tests should be described solely by name; describe more complex techniques in the Methods section.*
☐	☒	A description of all covariates tested
☐	☒	A description of any assumptions or corrections, such as tests of normality and adjustment for multiple comparisons
☐	☒	A full description of the statistical parameters including central tendency (e.g. means) or other basic estimates (e.g. regression coefficient) AND variation (e.g. standard deviation) or associated estimates of uncertainty (e.g. confidence intervals)
☐	☒	For null hypothesis testing, the test statistic (e.g. *F*, *t*, *r*) with confidence intervals, effect sizes, degrees of freedom and *P* value noted<br>*Give P values as exact values whenever suitable.*
☒	☐	For Bayesian analysis, information on the choice of priors and Markov chain Monte Carlo settings
☒	☐	For hierarchical and complex designs, identification of the appropriate level for tests and full reporting of outcomes
☒	☐	Estimates of effect sizes (e.g. Cohen's *d*, Pearson's *r*), indicating how they were calculated

Our web collection on statistics for biologists contains articles on many of the points above.

Software and code

Policy information about availability of computer code

Data collection	N/A
Data analysis	Sequence reads were aligned to the GRCh37 reference genome using BWA (v0.5.7). Reads were discarded based on mapping quality or if they failed the Illumina chastity filter and duplicate reads were marked using Picard (v1.11). Gene level coverage analysis was performed using the BCGSC pipeline v1.1 with Ensembl v69 annotations and were normalized based on RPKM (reads per kilobase per million mapped reads) or TPM (transcripts per million). No software was used for data collection. Principal component analysis (PCA) was performed using the prcomp R package, which is included R core base. PCA visualization was performed using the rgl (v0.100.54) R package. Nearest shrunken centroid analysis was performed using the pamr (v1.56.1) package. Uniform manifold approximation and projection (UMAP) was performed using the umap (v0.2.6.0) R package. Gene set enrichment analysis (GSEA) was performed using command line tools as previously described. Hierarchical clustering was performed using the made4 (v.1.58.0) R package. Circos figures were generated using the Circos (v0.69-9) software package. To extract a core subset genes from LSC47 that best explained patient outcomes in the training cohort, we used the same linear regression technique that formed the basis of LSC17 and is based on the LASSO (least absolute shrinkage and selection operator) algorithm as implemented in glmnet (v4.0-2) R package. Fusion calls were made using CICERO (v1.8.1), STAR-Fusion (v.1.10.1), and Trans-ABySS (v.2.0.1). Box plots were generated using ggplot2 (v.3.3.5).

For manuscripts utilizing custom algorithms or software that are central to the research but not yet described in published literature, software must be made available to editors and reviewers. We strongly encourage code deposition in a community repository (e.g. GitHub). See the Nature Portfolio guidelines for submitting code & software for further information.

Data

Policy information about availability of data

All manuscripts must include a data availability statement. This statement should provide the following information, where applicable:

- Accession codes, unique identifiers, or web links for publicly available datasets
- A description of any restrictions on data availability
- For clinical datasets or third party data, please ensure that the statement adheres to our policy

The data generated for this study have been deposited in dbGaP (https://www.ncbi.nlm.nih.gov/gap) under the dbGaP study ID phs000465.v21.p8 (https://www.ncbi.nlm.nih.gov/projects/gap/cgi-bin/study.cgi?study_id=phs000465.v21.p8) and in the TARGET Data Matrix at the TARGET Data Coordinating Center (https://target.nci.nih.gov/dataMatrix/TARGET_DataMatrix.html). TCGA AML (LAML) RNA-sequencing data was downloaded from the Broad Institute GDAC Firehose repository. The GRCh37 reference genome is available for download through the Michael Smith Genome Science Centre (https://www.bcgsc.ca/downloads/genomes/9606/hg19).

Field-specific reporting

Please select the one below that is the best fit for your research. If you are not sure, read the appropriate sections before making your selection.

[x] Life sciences [ ] Behavioural & social sciences [ ] Ecological, evolutionary & environmental sciences

For a reference copy of the document with all sections, see nature.com/documents/nr-reporting-summary-flat.pdf

Life sciences study design

All studies must disclose on these points even when the disclosure is negative.

Sample size	Sample size was determined based number of available diagnostic patient samples that underwent RNA sequencing.
Data exclusions	No data was excluded.
Replication	Our cohort was divided into training and validation cohorts. Novel risk model building (LSC47) was performed using our training cohort and our finalized risk model was assessed using our validation cohort. In addition, the risk stratification model used within our initial training and validation cohorts was further validated in an independent cohort in response to reviewer critiques. Within all three cohorts, LSC47 outperformed a previously validated risk prediction model (LSC17) based on significant differences in survival within AML subtypes (which was not observed with LSC17), area under the curve receiver operating characteristic curves (comparing LSC47 with LSC17), and multivariate hazard ratio survival analysis (which included LSC47 and LSC17 among other covariates).
Randomization	We performed stratified randomization based on underlying driver fusion to separate our cohort into training and validation (1:1). This allowed us to ensure that each cohort had similar representation of underlying driver fusion.
Blinding	Investigators were blinded to the validation cohort during risk model building. Group allocation was stratified based on fusion status, but was otherwise performed in an unbiased, automated manner by otherwise assigning patients into training and validation cohorts in alternating fashion on the basis of enrollment order.

Reporting for specific materials, systems and methods

We require information from authors about some types of materials, experimental systems and methods used in many studies. Here, indicate whether each material, system or method listed is relevant to your study. If you are not sure if a list item applies to your research, read the appropriate section before selecting a response.

Materials & experimental systems

n/a	Involved in the study	
[x]	[ ]	Antibodies
[x]	[ ]	Eukaryotic cell lines
[x]	[ ]	Palaeontology and archaeology
[x]	[ ]	Animals and other organisms
[ ]	[x]	Human research participants
[ ]	[x]	Clinical data
[x]	[ ]	Dual use research of concern

Methods

n/a	Involved in the study	
[x]	[ ]	ChIP-seq
[x]	[ ]	Flow cytometry
[x]	[ ]	MRI-based neuroimaging

Human research participants

Policy information about studies involving human research participants

Population characteristics	Pediatric AML biological samples were collected with informed consent from patients enrolled on Children’s Oncology Group (COG) trials CCG-2961, AAML03P1, AAML0531, or AAML1031. Enrolled patients were diagnosed with de novo AML and were 0 to 29 years of age at the time of diagnosis. Specific patient characteristics are described in Table 1 of our manuscript: gender, age, WBC count at diagnosis, cytomolecular risk group, MRD at the end of induction, stem cell transplant in first complete remission, mutational status, and fusion status.
Recruitment	Since enrollment eligibility was based on patients presenting with de novo AML and all sites were encouraged to offer enrollment to all eligible patients, no selection bias is anticipated. Clinical trials in acute leukemia are unique since the diagnosis itself (acute leukemia) represents an oncologic emergency. Therefore, the recruitment process is straightforward: eligible patients were offered study enrollment at participating sites upon initial presentation and diagnosis. Other traditional clinical trial recruitment procedures (e.g., advertising) are neither applicable nor ethical since initiation of therapy is required within hours to a few days of initial presentation.
Ethics oversight	Pediatric Central IRB. Additionally, all patients were enrolled and treated on one of the previously mentioned Children's Oncology Group clinical trials opened under IRB approval at each participating institution. Local IRB approval for each study was obtained by each site prior to enrolling patients. Sites were required to submit IRB approvals to the NCI’s Cancer Trials Support Unit (CTSU) Regulatory Office prior to enrolling patients.

Note that full information on the approval of the study protocol must also be provided in the manuscript.

Clinical data

Policy information about clinical studies

All manuscripts should comply with the ICMJEguidelines for publication of clinical research and a completedCONSORT checklist must be included with all submissions.

Clinical trial registration	CCG-2961 (NCT00002798), AAML03P1 (NCT00070174), AAML0531 (NCT00372593), AAML1031 (NCT01371981)
Study protocol	Available upon request
Data collection	Data was collected by each participating institution and aggregated by the Children's Oncology Group. Dates of accrual are as follows: CCG-2961/NCT00002798 (08/30/1996-06/16/2003), AAML03P1/NCT00070174 (12/29/2003-11/01/2005), AAML0531/NCT00372593 (08/14/2006-06/15/2010), AAML1031/NCT01371981 (06/20/2011-07/31/2017)
Outcomes	Primary outcome measures were event free survival and overall survival, which were assessed based on time to relapse/death and death, respectively.
